# *In Silico* Analysis Identifies Intestinal Transit as a Key Determinant of Systemic Bile Acid Metabolism

**DOI:** 10.3389/fphys.2018.00631

**Published:** 2018-06-08

**Authors:** Fianne L. P. Sips, Hannah M. Eggink, Peter A. J. Hilbers, Maarten R. Soeters, Albert K. Groen, Natal A. W. van Riel

**Affiliations:** ^1^Department of Biomedical Engineering, Eindhoven University of Technology, Eindhoven, Netherlands; ^2^Department of Endocrinology and Metabolism, Academic Medical Center, Amsterdam, Netherlands; ^3^Department of Vascular Medicine, Academic Medical Center, Amsterdam, Netherlands; ^4^Department of Laboratory Medicine, University of Groningen, University Medical Center Groningen, Groningen, Netherlands

**Keywords:** mathematical modeling, postprandial bile acid metabolism, intestinal transit, plasma bile acids, postprandial signaling

## Abstract

Bile acids fulfill a variety of metabolic functions including regulation of glucose and lipid metabolism. Since changes of bile acid metabolism accompany obesity, Type 2 Diabetes Mellitus and bariatric surgery, there is great interest in their role in metabolic health. Here, we developed a mathematical model of systemic bile acid metabolism, and subsequently performed *in silico* analyses to gain quantitative insight into the factors determining plasma bile acid measurements. Intestinal transit was found to have a surprisingly central role in plasma bile acid appearance, as was evidenced by both the necessity of detailed intestinal transit functions for a physiological description of bile acid metabolism as well as the importance of the intestinal transit parameters in determining plasma measurements. The central role of intestinal transit is further highlighted by the dependency of the early phase of the dynamic response of plasma bile acids after a meal to intestinal propulsion.

## Introduction

Bile acids are amphipathic cholesterol metabolites that fulfill a broad variety of metabolic functions (Lefebvre et al., 2009). Historically, bile acids have mainly been studied for their pivotal role in facilitating digestion and absorption of dietary lipids from the intestinal lumen (Lefebvre et al., 2009; Hofmann and Hagey, 2014). Additionally, bile acids have been found to interact with the microbiome (Ridlon et al., 2014), to constitute a major route of cholesterol excretion from the body (Staels and Fonseca, 2009), and to affect glucose and lipid metabolism in a hormone-like fashion via the Takeda G protein-coupled receptor 5 (TGR5) and the farnesoid X receptor (FXR) (Lefebvre et al., 2009; van Nierop et al., 2017). Moreover, bile acids are potential postprandial signals, as they are released in response to a meal. These functions place bile acids at the crossroads of digestion, metabolic regulation, and cholesterol homeostasis.

Consequently, the involvement of bile acids in obesity and its associated metabolic diseases is under investigation (Prawitt et al., 2011; Li and Chiang, 2015). Obesity itself is associated with elevated bile acid synthesis markers (Steiner et al., 2011; Haeusler et al., 2016). Insulin resistance and Type 2 Diabetes Mellitus, moreover, have been linked to an increased fasting plasma concentration in combination with shifts of the conjugation and composition of plasma bile acids (Haeusler et al., 2013; Wewalka et al., 2014; Sonne et al., 2016). The postprandial response of plasma bile acids is also implicated in metabolic disease, as it is blunted in obesity (Suzuki et al., 2014; Haeusler et al., 2016), but amplified following bariatric surgery (Cole et al., 2015; Penney et al., 2015). However, the role bile acids play in these cases remains unclear.

Understanding how underlying processes such as synthesis or intestinal transit and re-uptake quantitatively underlie fasting and postprandial bile acid measurements is critical to resolving the involvement of bile acids in metabolic health. Measurements of bile acids in non-portal plasma, on which bile acid research predominantly relies (Ma and Patti, 2014), only partially reflect enterohepatic bile acid concentrations. Due to rapid differential hepatic clearance, bile acid concentrations in plasma are low and the composition of the plasma pool does not mirror the composition of bile acids elsewhere (Hofmann and Hagey, 2014; Eggink et al., 2017). Furthermore, due to the accumulation of bile acids in the body, the rapid enterohepatic circulation (EHC) and the continual liver and intestinal transformations, propagation of local perturbations to plasma measurements is complex. Therefore, although many of the transporters and processes responsible for plasma bile acid excursions have been elucidated (LaRusso et al., 1978; Dawson et al., 2009), it remains difficult to understand the postprandial dynamics of individual bile acid species (Steiner et al., 2011; Zhang et al., 2011; Fiamoncini et al., 2017).

To gain insight into the major determinants of plasma bile acid measurements and postprandial responses in health, we developed a mathematical model of bile acid metabolism. The model describes the enterohepatic and peripheral circulation of bile acids via differential equations and thus relates bile acid concentrations in the plasma pool to bile acid metabolism and concentrations in tissues throughout the EHC. We found that a distal slowing and a postprandial increase of intestinal transit speed were important elements of the model in order to describe the postprandial response. Furthermore, the postprandial response was found to consist of three distinct phases, governed by intestinal propulsion, primary gallbladder emptying and the recycling of bile acids, respectively. Finally, we performed a quantitative analysis of the relation between plasma measurements and model parameters, which revealed proximal, distal and colonic transit as prominent, but contrasting, determinants of plasma bile acid concentrations.

## Materials and Methods

### Mathematical Model

**FIGURE 1:**
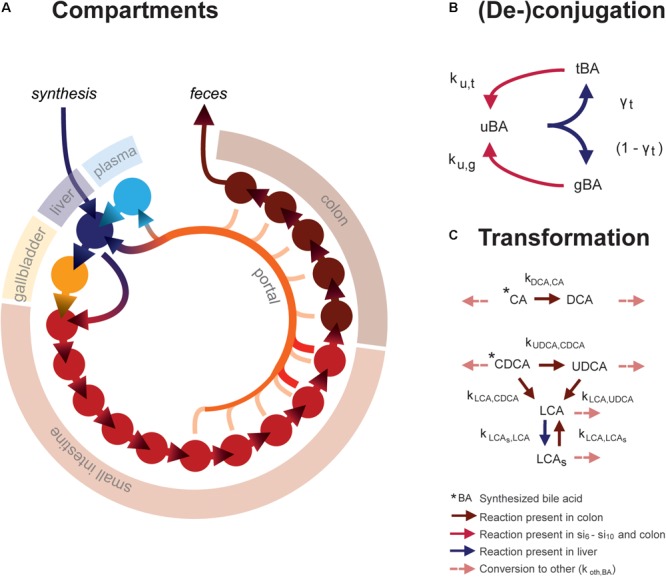
Mathematical model. **(A)** Overview of model compartments and transportation. Note that the plasma compartment is not a component of the enterohepatic circulation (EHC), and only bile acids that do not enter the (intracellular) liver compartment spill over into the plasma compartment. **(B)** Model reactions describing bile acid conjugation from unconjugated (*uBA*) to either taurine (*tBA*) or glycine (*gBA*) conjugated BA and respective deconjugation. Arrow colors denote the compartments in which reactions take place, as described in the legend. **(C)** Bile acid transformations. For simplicity, other bile acids (o) are not pictured. Note that transformation only takes place in the colon, in contrast to deconjugation, which can take place in the final five small intestinal compartments as well as the colon.

The ordinary differential equation (ODE) model describes the metabolism and conversion of bile acids in a cycle of connected compartments that represent the EHC (**Figure 1A**). Bile acids enter the cycle exclusively in the liver compartment (*li*) via synthesis, before being rapidly excreted from the liver into either the first intestinal compartment (*si*_1_) or the gallbladder compartment (*gb*) for storage. Gallbladder emptying into *si*_1_ undergoes fasting-feeding cycles; emptying is low during fasting and high following feeding. Intestinal transportation and uptake of bile acids is modeled with a series of 15 intestinal compartments, which represent the duodenum (*si*_1_), the jejunum (*si*_2_ -*si*_5_), the ileum (*si*_6_ -*si*_10_) and the colon (*co*_1_ - *co*_5_).

The model distinguishes between the most common human bile acids, and so describes cholic acid (CA), chenodeoxycholic acid (CDCA), deoxycholic acid (DCA), ursodeoxycholic acid (UDCA), litocholic acid (LCA), as well as a generic “other” (o) bile acids class, as separate species. Additionally, it includes the sulfated form of LCA (LCA_s_), as a large portion of plasma LCA are sulfated (in contrast to other bile acids, of which only a small portion is sulfated), and because sulfation reduces the efficiency of intestinal bile acid uptake and can be an important route of bile acid excretion (Alnouti, 2009). Transformation of bile acids, as is mediated by microbiota *in vivo*, takes place in the colon (**Figure 1C**). Additionally, the model comprises unconjugated, taurine- and glycine-conjugated forms of each bile acid, which will be further denoted by prefixes *u, t*, and *g*. Unconjugated bile acids are absorbed from the intestines via passive uptake along the entire length of the intestine. Active uptake of conjugated and unconjugated bile acids takes place only in the two terminal compartments of the small intestine (*si*_9_ and *si*_10_). Bile acids in the terminal small intestine and colon undergo deconjugation.

Intestinal transit is included in a temporally and spatially heterogeneous way. Proximal small intestinal transit is fast (k_si,α_) whilst terminal transit is slow (k_si,β_) – but not as slow as colonic transit (*k*_co_). In response to a meal, transit speed is transiently increased, propelling the intestinal contents forward. Following intestinal uptake, recycled bile acids either return directly to the liver via first pass clearance or enter the plasma compartment (*pl*). Hepatic extraction – the ratio of the bile acids taken up to the total amount of bile acid passing through the liver – is governed by the bile acid-specific parameters ψ_tri_, ψ_di_, ψ_mono_, ψ_u_, and ψ_sulf_. Here, the subscripts refer to the number of OH-groups and molecular structure of the bile acid, and conjugation or sulfation status. This is necessary to account for differences in hepatic extraction between bile acids of different molecular structures. Extraction is more efficient for conjugated than unconjugated bile acids (Dawson et al., 2009; Hofmann and Hagey, 2014; Eggink et al., 2017). Molecular structure affects bile acid extraction as a result of differences in affinity of the transporters responsible for hepatic uptake – the Na^+^- taurocholate co-transporting polypeptide (NTCP) and members of the organic anion transport (OATP) family (Suga et al., 2017).

Altogether, the model thus describes the kinetics of 21 bile acid configurations in 18 compartments (li, gb, pl, si_1_, si_2_, … si_10_, and co_1_, co_2_, … co_5_). To reach a fasting configuration, the ordinary differential equation model is simulated for a number of days. On each day, three meals are simulated with intervals of 6, 6, and 12 h to complete a 24-h day. In this way, a dynamic state is reached in which the fasting condition at the beginning of each day is the same as the day before. Full model equations are provided in the Supplementary Data S1 and Supplementary Tables S1, S2.

### Calibration Dataset

**FIGURE 2:**
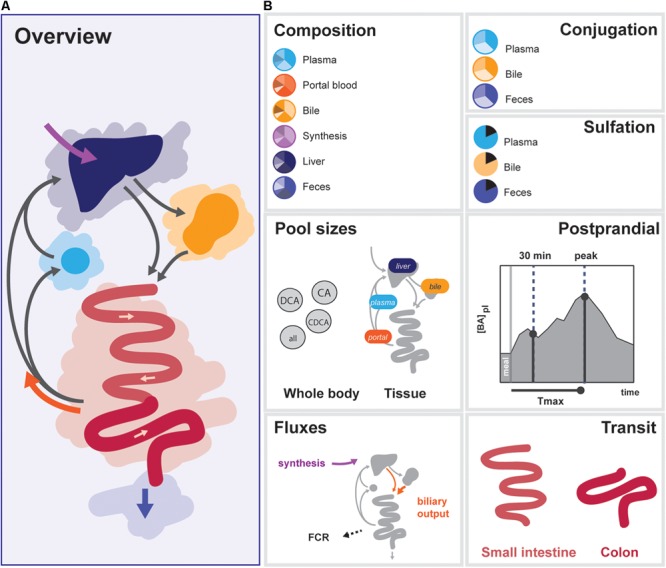
Overview of data included in the CDS. **(A)** Overview of the enterohepatic circulation (EHC). **(B)** Components of the CDS: (1) Composition of the bile acid pool in various tissues, (2) conjugation of bile acids in plasma, bile and feces, (3) Sulfation of LCA in bile and plasma, total sulfation in plasma and feces, (4) Organ (plasma, portal blood, liver, and gallbladder) and bile acids species-based (CA, CDCA, DCA, and total) pools, (5) Postprandial characteristics (30 min increase, maximal increase, and time of the maximal increase) of total bile acids, conjugated bile acids and unconjugated bile acids in plasma, (6) Fluxes (synthesis, biliary output, and fractional catabolic rate), (7) Small intestinal transit after a meal and during fasting, and colonic transit.

In order to determine the 33 free model parameters and evaluate the ability of the model to describe bile acid metabolism, a composite calibration dataset (CDS) was composed from the literature. Data was included in the CDS based on the following criteria: (1) the data was measured *in vivo* or via biopsy directly in humans, (2) three or more independent repetitions were reported in the literature, and (3) either the individual data or mean values and sample sizes were reported. A total number of 99 studies were included. To obtain a coherent dataset, the data was then converted to uniform units and a mean over all individuals was calculated, resulting in 76 individual data points (Supplementary Data S1). The CDS can globally be divided into 7 datatypes representing different aspects of intestinal transit and bile acid metabolism – composition, conjugation, sulfation, pool sizes, fluxes, postprandial measurements, and intestinal transit times (**Figure 2**).

### Parameter Estimation

To find a set of parameters for which the model describes the CDS well, the normalized sum of squared errors nSSE was minimized (Equation 1)

(1) $$\begin{array}{c} nSSE(p)\;=\;\sum_{i\;=\;1}^{N_{CDS}}{\left(w_{i}\cdot \frac{y_{i}(p)-d_{i}}{d_{i}}\right)}^{2} \end{array}$$

Here, ***p*** is the vector of model parameters, *N*_CDS_ is the number of data points in the CDS, *d*_i_ is the ith data point in the CDS, y_i_ (***P***) is the corresponding model output, calculated with parameter vector ***p*** in the dynamic steady state described above. *w*_i_ is the weight for the current data point, which is 1 for all data points in the pool sizes, fluxes, postprandial characteristics and transit categories, but weighs the number of species included in the current composition in the composition, conjugation, and sulfation categories. This prevents overemphasizing composition, conjugation and sulfation data in the calibration procedure (Supplementary Data S1). The optimal parameter set fulfills

(2) $$\begin{array}{c} p_{opt}\;=\;\arg\min_{p\;\in \;phr}\;nSSE(p) \end{array}$$

i.e., the normalized cost function is minimized for ***p***, where the values of ***p*** are bounded in their chosen (physiological) ranges (***phr***).

### Model Validation

#### Estimates of Hepatic Extraction Parameter Values

Estimates of parameter values of hepatic extraction were calculated from two sources. First, ψ_tri_, ψ_di_, ψ_mono_, and ψ_u_ were calculated from portal and venous fasting bile acid concentrations obtained from human subjects described in (Eggink et al., 2017). Second, ψ_u_ was also calculated from postprandial arterial, portal and hepatic venous bile acid concentrations obtained from pigs (Schooneman et al., 2015; Eggink et al., 2017). For further details, we refer to Supplementary Data S1.

#### Systemic Fluxes

Systemic fluxes over 24 h were calculated and expressed relative to the 24 h secretion, which was defined as the total amount of bile acids secreted into the duodenum (*si*_1_) from both the liver directly and the gallbladder over 24 h.

#### *In Silico* Cholecystectomy

To simulate cholecystectomy, the gallbladder is simply taken out of the system by setting the fraction of hepatic output that is sent to the gallbladder (γ_gb_) to 0.

### Sensitivity, Identifiability and Necessity of Model Components

To investigate the uncertainty in parameter values and model predictions, three strategies were employed: (1) the sensitivity of the nSSE to the parameter values was determined (*S*_nSSE_), (2) the necessity of several model components was evaluated by removing parameters and equations from the model, and (3) a profile likelihood (Raue et al., 2009) was generated. The profile likelihood is performed to assess per parameter how well this parameter is constrained by the data. A profile is created for each parameter by progressively fixing this parameter further and further from its optimal value and then reoptimizing all remaining parameters, to obtain a minimal value of nSSE given the fixed parameter. The result is a relationship between the value of a parameter and the corresponding minimal possible value of nSSE. In the case this relationship is very steep, i.e., a small change of the parameter value will cause a large increase of the nSSE, the parameter in question is deemed identifiable from the data. A (partially) flat profile, conversely, signifies that based on the available information the parameter’s value may lie anywhere in such a flat region while the description of the data is (practically) unaffected.

### Analysis of the Postprandial BA Response

To gain more insight into the processes underlying the postprandial response, we calculated the postprandial response with regular parameter values (***p***_**normal**_), with the postprandial increase in intestinal propulsion δ_SI_ set to 0 (***p***_**δ**_**SI**_**=0**_), and with the liver-mediated recycling disabled (***p***_**k**_**xl**_**=0**_).

### Local Parameter Sensitivity Analysis

The effect of a parameter *p*_k_ on a plasma measure – the sensitivity *S*_PM_ – was calculated to give insight into the factors that have the most influence on, e.g., postprandial measurements of plasma bile acids. The local parameter sensitivity analysis was performed by applying a small change to the value of a single parameter while keeping all others fixed, and evaluating the resulting change in some model outcome, represented by a (vector) function ***f*** (of length *N*_f_). Each parameter *p*_k_ is sequentially changed by a factor of Δp_k_.

(3) $$\begin{array}{c} S_{PM}{}_{p_{k}}^{f}\;=\;\frac{1}{2△N_{f}}\sum_{j=1}^{N_{f}}\frac{\left|f_{j}(p_{k})-f_{j}(p_{k}+△\cdot p_{k})|+|f_{j}(p_{k})-f_{j}(p_{k}-△\cdot p_{k})\right|}{f_{j}(p_{k})} \end{array}$$

### Software and Implementation

The model was implemented in Matlab (2012a/2016b, The MathWorks, Natick, MA, United States). The differential equations were solved using MEX-files compiled with the aid of the SUNDIALS CVode package (2.6.0, Lawrence Livermore National Laboratory, Livermore, CA, United States) (Hindmarsh et al., 2005; Vanlier et al., 2012). For parameter estimation, non-linear least squares optimizer LSQNONLIN was used as described above. Further details on model optimization and simulation, calculation of output variables, and sensitivity analyses, as well as an implementation of the model in Matlab, can be found in Supplementary Datas S1, S2.

## Results

The following sections describe the constructed mathematical model and collection of data used for model calibration (calibration dataset, or CDS), model validation, evaluation of parameter sensitivity and identifiability, and finally model analysis via decomposition of the postprandial response and sensitivity and control analyses.

### Mathematical Model and Calibration

The mathematical model (**Figure 1**) describes the EHC of all major types of human bile acids. In total, the model encompasses 6 bile acid species and one sulfated bile acid species, each of which are described in 3 conjugation states, in each of the model’s 18 compartments. The model contains 33 free parameters, the values of which were determined by fitting the model to the 76 datapoints of the CDS (**Figure 2** and Supplementary Figures S1, S2).

The CDS combines data from the literature (Supplementary Table S3) and combines information about the nature of the bile acid pool in a number of compartments. More specifically, it encompasses composition, conjugation, and sulfation measurements in the (non-portal) plasma pool, the portal blood, the liver pool, the newly synthesized bile acid flux, the gallbladder pool and the feces. Also, the dataset includes information about the size and distribution of the bile acid pool. This includes measurements of the total bile acid pool, the total pools of CA, CDCA, and DCA, and the pools present in plasma, portal blood, liver, and gallbladder. The CDS also encompasses direct measurements of fluxes in the model, including bile acid synthesis, measurements of the biliary bile acid output and fractional catabolic rates of the primary bile acids. Furthermore, several general postprandial characteristics were included: the time of the postprandial peak, the relative height of the postprandial peak and the relative height at 30 min after a meal. Finally, the CDS contains measurements of fasting and postprandial small intestinal and colonic transit speeds.

The final composite dataset thus consists of 76 individual values, which we globally divide into seven categories of bile acid composition, conjugation status, sulfation status, pool sizes, fluxes, and the postprandial characteristics of bile acid metabolism, as well as intestinal transit times (**Figure 2**). The dataset consists entirely of measurements performed in humans, and provides a quantitative summary of bile acid metabolism to which parameters can be estimated and model performance can be evaluated. Following parameter estimation, the calibrated model was found to reproduce the CDS well both qualitatively and quantitatively (see Supplementary Figures S2, S6).

### Validation

Model validation was performed by (1) comparing model parameters against values calculated directly from independent experimental data (2) evaluating variables and dynamics not included in – and with a different nature to – the CDS, (3) predicting the bile acid response to a change of physiology – namely an *in silico* cholecystectomy and (4) analysis of determinants of the pool composition.

#### Parameter Values

Direct estimations of hepatic extraction (HE) ratios were calculated from measurements of plasma and portal bile acid concentrations and compared with the estimated parameter values. Model parameters yielded good agreement with values calculated from experimental results both quantitatively and qualitatively. The value of tri-hydroxylated bile acid extraction (ψ_tri_) of 0.95 closely resembles the experimental value of 0.97 ± 0.005 (mean ± SEM), and the value of ψ_di_ (0.84) is almost identical to the experimental value of 0.82 ± 0.07. Unlike ψ_tri_ and ψ_di_, the value of ψ_mono_ was found to be practically unconstrained by the model (see Supplementary Figure S4); its range is in good agreement with the experimentally determined 0.64 ± 0.08. Finally, the ratio between HE of conjugated and unconjugated bile acids ψ_u_, which rests at its literature-derived lower bound of 0.625 in the optimized model, corresponds well with the new estimates obtained from human (0.59 ± 0.08) and porcine (0.65 ± 0.02) measurements.

#### Independent Variables and Dynamics

**FIGURE 3:**
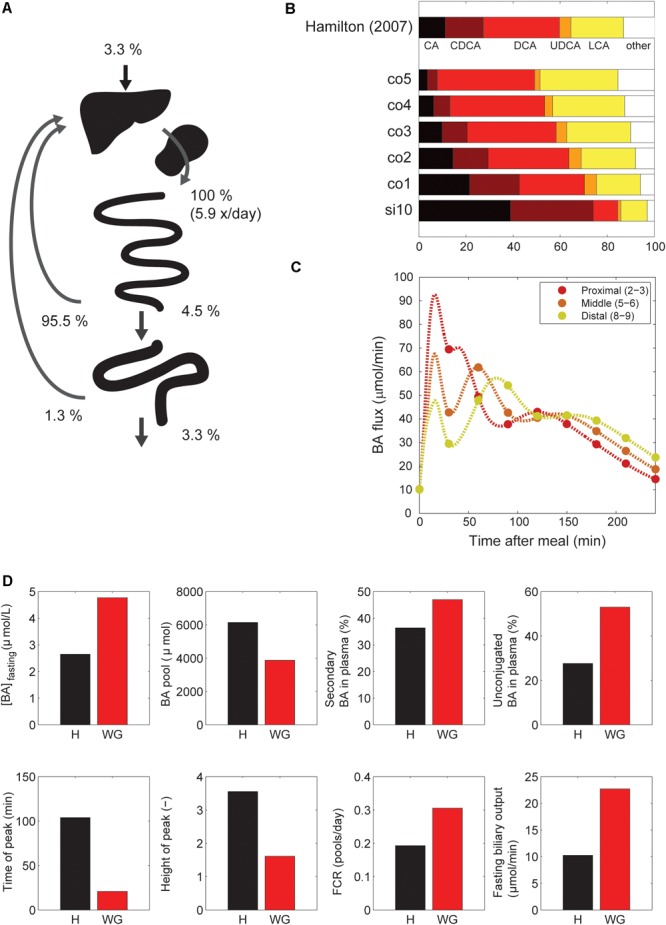
Model validation. **(A)** Overview of the main whole-body level fluxes of total bile acids, expressed in % of bile acids secreted into the duodenum (100% is thus defined as the total flux into the duodenum from liver and gallbladder, which equates to the total bile acid pool being excreted 5.9 times per day). Also visualized are the flux from small intestine to the colon, the flux from the colon to the feces, synthesis, and the total amount of bile acids recycled from small intestine and colon. Note that as these fluxes represent net recycling, temporary storage of bile acids in plasma or gallbladder is not pictured. **(B)** Composition in terminal ileum compartment and each colon compartment, and data of the cecum bile acid composition as reported in Hamilton et al. (2007). **(C)** Flux between 2nd and 3rd, 5th and 6th and 8th and 9th compartments of the small intestine. **(D)** Simulations of the normal model vs. *in silico* cholecystectomy (without gallbladder – WG). The normal model is visualized in black and the model without a gallbladder in red.

Next, we examined the major systemic bile acids fluxes (**Figure 3A**), cecal bile acid compositions (**Figure 3B**) and measurements of dynamic intestinal fluxes (LaRusso et al., 1978) (**Figure 3C**).

A well-known characteristics of bile acids is their efficient recycling within the EHC (Hofmann and Hagey, 2014). For instance, less than 5% of the secreted bile acids passes into the colon, and an even smaller fraction is lost in the feces (Chiang, 2009). The *in silico* proportion of secreted bile acids to reach the colon (4.5%) is thus in good agreement with the literature (**Figure 3A**), as is the proportion (3.3%) of secreted bile acids lost in feces.

The composition of the bile acid pool in the colon was compared with cecal bile acid composition as previously analyzed in deceased subjects (Hamilton et al., 2007) (**Figure 3B**). The composition of bile acids in the cecum closely resembled the *in silico* composition in the second colon compartment. The simulations of intestinal fluxes of bile acids (**Figure 3C**) also showed good qualitative as well as quantitative agreement with the experimental data, and confirmed that *in silico*, as *in vivo*, bile acids are propelled deep into the intestine immediately following a meal. Finally, the computation of a tracer ([75 Se] HCAT) half-life yielded a faster turnover of tracer in the model than is commonly found *in vivo* (see Supplementary Table S4). Since kinetics of several bile acid species were included in the CDS and were accurately described by the model, this may point to an inaccurate description of tracer handling, or a more generic overestimation of the synthesis rate as included in the CDS.

#### *In Silico* Cholecystectomy

The cholecystectomy is a common surgical procedure of which the effect on bile acid metabolism has been studied extensively (Berr et al., 1989; Kullak-Ublick et al., 1995). With our model, we simulated only the gallbladder removal itself (**Figure 3D**) without any further parameter changes, and so do not account for any adaptations that may take place *in vivo*. Interestingly, the qualitative changes we observed in the simulation were in agreement with literature data (Supplementary Table S4). The postprandial response, for instance, is conserved when the gallbladder is lost, although the peak is commonly found earlier and has a lower amplitude – characteristics which were all reproduced by the model.

Finally, we applied the model to predict the major determinants of the DCA pool *in vivo.* We choose to predict the determinants of the DCA pool, as the influence of intestinal transit speed on DCA pool size has been demonstrated in literature (Marcus and Heaton, 1986; Veysey et al., 2001), and the *in silico* reproduction of such an experiment was used as a validation of the control and sensitivity analyses. The distal intestinal transit parameters were indeed predicted to be major determinants of the DCA pool *in silico* (see Supplementary Figure S3).

### Uncertainty Analysis

Sensitivity of the model to parameter values was assessed via a local sensitivity analysis (see Supplementary Figures S4, S5). Further diagnostics of parameter uncertainty were performed via a profile likelihood analysis (see Supplementary Figure S4). Additionally, to evaluate model complexity, we analyzed the necessity of the spatial and temporal complexity of the intestinal propulsion in the model and showed both were necessary. See Supplementary Figure S6.

### Postprandial Response

**FIGURE 4:**
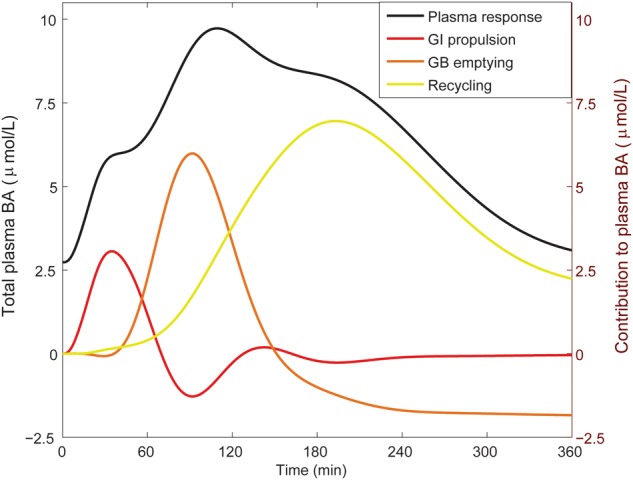
Components of the postprandial BA response in plasma. The total bile acid response in plasma after a meal is shown (black; left axis), along with its decomposition (right axis) in a component dominated by gastro-intestinal (GI) propulsion (red), a component composed of bile acids that have been recycled through the liver since the start of the meal (yellow), and the final dynamic component, which is calculated by subtracting the two previous components from the total normalized bile acid response, and reflects primary gallbladder (GB) emptying (orange). These components represent the change from the fasting concentration driven by the respective process. The GI propulsion and recycling components are calculated by disabling increased GI propulsion or hepatic recycling and calculating the difference to the normal response. The remaining change of plasma concentration is allocated to gallbladder emptying alone to reflect the fact that the major dynamic change captured in this component clearly stems from gallbladder emptying, although strictly speaking this curve combines all other model components. Note that the contributions may become negative, when the predicted plasma concentration is below 0 (but the sum of all three is equal to the total change from fasting). The negative contribution from (primary) gallbladder emptying represents the decrease of plasma bile acids after the meal when there is no recycling – i.e., the gallbladder does not refill.

From the literature (see CDS), we characterized the nature of the transient increase of plasma bile acid concentrations after a meal. This increase is usually initiated shortly after food intake and so is already apparent 30 min after a meal, but does not reach its maximal value until it culminates in a peak at around 90 min. The maximal concentration of total bile acids is over three times the fasting concentration. After fitting to the postprandial characteristics included in the CDS (**Figure 4**), this postprandial response is indeed reproduced by the model. To better understand underlying mechanisms, we sequentially disabled model components, until we found the postprandial increase to be composed of three main phases. The first phase spans the first hour after a meal and is the result of postprandially increased intestinal propulsion. The second phase contains a peak (at around 80 min) that reflects the emptying of the contents stored in the gallbladder in the fasting state. Finally, the third phase, which has a late peak at about 150 min, consists of bile acids that quickly cycled after the meal, and – since the gallbladder is in a postprandial state – are not stored, but quickly re-secreted. As a result of this final phase the entire bile acid pool is secreted more than once per meal (Hofmann and Hagey, 2014).

### Determinants of Plasma Bile Acids

**FIGURE 5:**
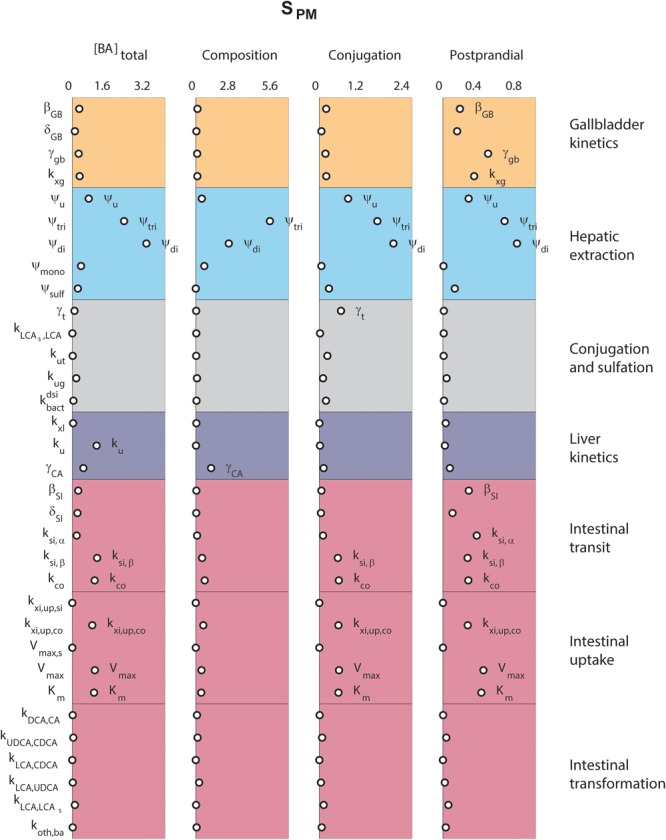
Determinants of plasma bile acids. Sensitivity analyses were performed to determine the main determinants of four different plasma characteristics: (left) fasting total plasma bile acid concentration; (second column) the composition of the five main bile acids, in non-sulfated form; (third column) the conjugation of plasma pool; (right) the relative postprandial response of the total plasma bile acid concentration. The 33 parameters are distributed along the vertical axis. For full descriptions of all parameters, we refer to the section “Materials and Methods.” In short, β and δ denote parameters of the postprandial dynamics, ψ are hepatic extraction parameters, γ are fractions, *k, V*_max_, and *K*_m_ are (mass action) rates and Michaelis–Menten parameters for bile acid transportation or conversion.

Finally, we analyzed the major determinants of plasma bile acid concentrations via a local sensitivity analysis (**Figure 5**). The resulting sensitivities, the *S*_PM_, are predictions of to what extent model parameters affect a projected outcome – in this case a plasma measurement of bile acids.

**Figure 5** compares sensitivities for four different plasma measures. The results show that some parameters, such as those governing conjugated bile acid hepatic extraction (ψ_tri_, ψ_di_), affect all plasma measures, whereas others affect some plasma measures, but not others. For instance, the synthesis rate (k_u_), only controls the fasting plasma bile acid concentration. This fasting concentration is further determined by colonic passive (k_xi,up,co_) and ileal active uptake kinetics (*V*_max_, *K*_m_) and distal intestinal transit rates (k_si,β_ and k_co_). The parameters governing the postprandial response, on the other hand, include postprandial dynamic (β_GB_ and β_SI_) and gallbladder filling parameters (γ_GB_ and k_xg_). A notable difference between determinants of fasting and postprandial concentrations is that while the proximal small intestinal transit (k_si,α_) is of little influence on the fasting concentration it *is* of influence on the shape of the postprandial response. This again points to the different roles of distal (k_si,β_) and proximal (k_si,α_) intestinal transit. This distinction, which we have shown to be a vital part of the model (see Supplementary Figure S6), allows bile acids to slow in the distal intestine for increased uptake.

**FIGURE 6:**
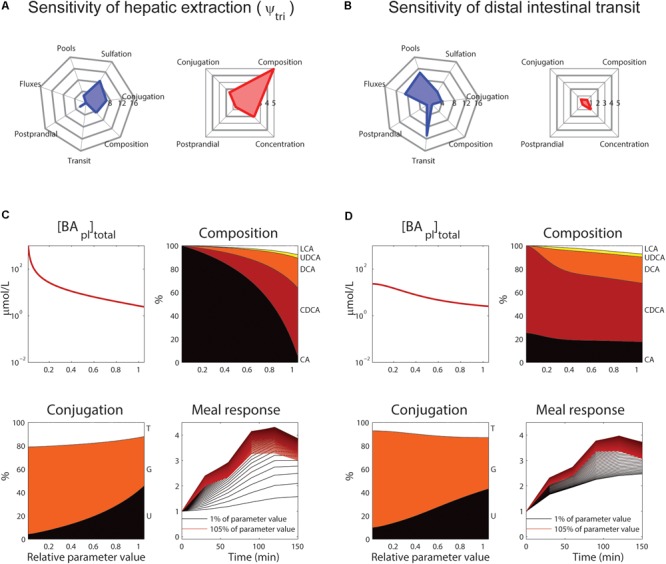
Control of hepatic extraction and distal small intestinal transit. **(A)** Spider plots of S_nSSE_ (left, sensitivity to the normalized sum of squared errors, which reflects how well the model describes the calibration dataset) and *S*_PM_ (right, the effect of a parameter on the plasma measures) for tri-hydroxylated bile acid extraction parameter ψ_tri_. In the spider plots, parameter sensitivities for different categories of nSSE (blue) and the four plasma measures as shown in **Figure 5** (red) are plotted radially. **(B)** As **(A)**, for distal intestinal transit parameter k_si,β_. **(C)** Control of parameter ψ_tri_ on total plasma concentration (top left), composition (top right), conjugation (bottom left), and postprandial response (bottom right). For the plasma concentration, composition, and conjugation, the *x*-axis represents the relative parameter value. In the composition and conjugation plots, the relative contributions of LCA (white), UDCA (yellow), DCA (orange), CDCA (red) and CA (black), or taurine-conjugated (T, white), glycine-conjugated (G, orange) and unconjugated (U, black) are displayed. The postprandial response plot, finally, shows the postprandial response for parameter values between 1 (black) and 105% (red) of the original value, with steps of 1%. **(D)** As **(C)**, for parameter k_si,β_.

The roles of tri-hydroxylated bile acid extraction (ψ_tri_) and distal small intestinal transit speed (k_si,β_) in bile acid metabolism are further illustrated in **Figure 6**. These parameters each affect how well the model describes multiple categories of the CDS (**Figures 6A,B**, blue spider plots), as well as multiple plasma measurements (**Figures 6A,B**, red spider plots). The direct influence of these two key parameters on fasting plasma concentration, composition, conjugation and the postprandial response is shown in **Figures 6C,D**.

## Discussion

In decades of bile acid research, the multi-faceted role of bile acids has caused interest in bile acid metabolism to repeatedly resurge and change focus (Hofmann and Hagey, 2014). Recently, the question of whether bile acids are involved in metabolic and postprandial signaling has reignited investigation of the postprandial plasma response of bile acids (Steiner et al., 2011; Sonne et al., 2013, 2016). Here, we examined the determinants of this response based on a mathematical model. By calibrating the model to a large calibration dataset, we not only obtained consistent values for model parameters, but we were also able to determine that a complex description of intestinal transit is necessary to unite model and data. The analysis of the final model revealed that the intricacies of intestinal transit are not only important for bile acid metabolism, but are also directly observable in the postprandial response of plasma bile acids and are amongst the most influential determinants of bile acid metabolism.

Previous mathematical models of bile acid metabolism have contributed to bile acid research. The series of models published by Hofmann et al. (1983, 1987), Molino et al. (1986), Cravetto et al. (1988) describe the EHC of CA, CDCA, and DCA, and especially the biliary handling, in great detail. More recent models (Woodhead et al., 2014; Edwards et al., 2016; Zuo et al., 2016) use a similar design to investigate the handling of LCA, UDCA, or the bile acid analog obeticholic acid, respectively. The model developed here differs from these in several important ways. First, as discussed above, the presented model describes the intestinal tract in more (spatial) detail. Also, the presented model includes all major human bile acids simultaneously, unlike the previously published models. Finally, we used a large, representative dataset from the literature (the CDS) to obtain estimates of parameter values that objectively merge quantitative data from the literature in an optimal way. The end result is a model that accurately describes postprandial responses and intestinal bile acid distributions and lends itself to investigation of metabolic signaling activity, for which the full composition of bile acids is essential (Vaquero et al., 2013).

In order to reproduce the CDS whilst obeying the restrictions imposed by bile acid physiology, we found the model required a complex description of intestinal transit – including both a temporal and a spatial dependency. Such complexity of intestinal transit results from the intricate regulatory system controlling intestinal motility. The described heterogeneity of transit has been previously described in the literature. The assumption that intestinal transit slows at the end of the ileum to facilitate uptake of fats and bile acids is supported by, e.g., the dying out of the majority of motor activity fronts in the intestine before reaching the terminal ileum (Seidl et al., 2012) and the delay observed in the ileocecal region when investigating intestinal transit (Pišlar et al., 2015). The transient acceleration of transit after a meal corresponds with meal-induced changes in intestinal motor pattern, as well as increases in transit trough the ileo-cecal junction and the colon, known as the gastro-ileocecal reflex and gastro-colonic response, respectively (Pišlar et al., 2015). Additionally, intestinal motility has been found to be controlled based on distal food and bile acid sensing (Hammer et al., 1998; Van Ooteghem et al., 2002). It is further supported by the difference in transit time between fasting and feeding states (Pišlar et al., 2015) and an immediate and seemingly uniform postprandial increase in small intestinal flux (Kerlin and Phillips, 1983). Evidence of non-homogeneous transit can be found in previous bile acid models (Hofmann et al., 1983, 1987; Molino et al., 1986; Cravetto et al., 1988), although it is implemented and analyzed here in more detail with the aid of the CDS.

It is well established that intestinal transit times affect bile acid metabolism (LaRusso et al., 1978) – e.g., the DCA pool increases in size when the colonic transit time is increased (Marcus and Heaton, 1986). However, the presented model allowed us to quantitatively dissect intestinal transit and explore its role in postprandial bile acid excursions in more detail. The results stress the role of the spatial and temporal non-homogeneity of intestinal transit for bile acid metabolism.

As is true for all models, there are inherent limitations to the applicability of the presented model stemming in part from the simplifications and choices made in its design. As already discussed, we chose to describe intestinal transit in detail, while describing other elements of the EHC more coarsely. Simplifications include the kinetics, as well as the absence of hepatic and biliary details. In addition, in the model active reabsorption of bile acids from the intestine, mediated by the apical sodium dependent bile acid transporter (ASBT), is localized only in the final two small intestinal compartments, as is distal (slow) transit. This localization of ASBT is based on expression profiles in humans well as animals (Stelzner et al., 2000; Dawson et al., 2009) and further supported by the sensitivity of bile acid metabolism to relatively small resections of the ileum (Walters et al., 2015). However, it would be reasonable to expect the transition from fast transit without active uptake to slow transit with active uptake to be more gradual *in vivo*. Furthermore, the model currently does not include a possible proximal active uptake mechanism (Dawson et al., 2009; Fiamoncini et al., 2017). However, data describing such a mechanism is sparse and a proximal uptake mechanism is thought to carry only a minor flux (Dawson et al., 2009), therefore we feel the presented model is an adequate approximation and allows quantitative analyses of general characteristics of bile acid metabolism.

In the analyses of postprandial responses, we have not looked beyond generic, averaged responses. However, it is important to note that the model can in the future also serve as a tool to investigate the large inter-individual differences (Steiner et al., 2011) and meal dependent changes in plasma bile acids (Sonne et al., 2016) of postprandial responses of bile acid metabolism. Gallbladder emptying, for instance, is determined by a number of meal characteristics such as caloric content, state (liquid or solid), and especially fat content (Marciani et al., 2013; Sonne et al., 2014). The meals included in this study are large, hypothetical meals and should be thought of as the result of dividing the total daily food intake into three identical sessions. Since the model is in no way restricted to this meal, the meal response can be straightforwardly adapted for a specific meal or for individual patients in future applications, allowing individualized modeling of bile acid metabolism.

To determine parameter values, we applied a composite calibration dataset, which guided the model development and provided us with a benchmark to compare the model to. In this approach, we did not employ measures of uncertainty of the data, as the main uncertainty propagated by the datasets lies not in the uncertainty in individual measurements, but stems from conflicts that come from combining data of many types and obtained via different methods. By combining these different types of data, we were able to balance possible biases in one measurement by other information. Ultimately, the model as calibrated on our CDS gives a complete picture of bile acid metabolism, as evidenced by the comparison to independent data.

As discussed briefly above, the comparison of the *in silico* cholecystectomy to an *in vivo* cholecystectomy situation is not complete, as it does not include adaptation after surgery. In this comparison, we – like Cravetto et al. (1988) – assumed that the source of postprandial excursions is proximal small intestinal bile acid storage (Said, 2012) due to continuous bile acid secretion (Bencze et al., 2012), as opposed to storage in a distended biliary system (Majeed et al., 1999). This assumption is supported by observations that duodenal bile acid concentrations do not display a postprandial rise after cholecystectomy (Sonne et al., 2013) and that bile acid output is not dependent on cholecystokinin in cholecystectomized patients (Malagelada et al., 1973). Also, biliary bile acid output after cholecystectomy – while increased in the basal state – only shows very mild changes postprandially, and only as a result of increased intestinal cycling (Nahrwold and Grossman, 1970). Compensatory adaptations to cholecystectomy *in vivo may* include changes of intestinal transit (Fort et al., 1996) and bile acid synthesis (Roda et al., 1978; Berr et al., 1989). These adaptations are implemented via several negative feedback mechanisms that control the size of the bile acid pool, such as regulation of bile acid synthesis via intestinal FXR signaling (Lan et al., 2013), regulation of the expression of transporters ASBT and NTCP, and control of intestinal transit (Van Ooteghem et al., 2002).

## Conclusion

Here we present a comprehensive *in silico* model of bile acid homeostasis. The model encompasses composition and postprandial kinetics, and is thus a good basis to obtain further mechanistic insight in bile acid perturbations in diseases state such as in type 2 Diabetes Mellitus (Sonne et al., 2016) or after bariatric surgery (Ahmad et al., 2013). Intriguing applications of the model lie in a personalized analysis of heterogeneous bile acid metabolism.

## Data Availability Statement

The model generated in this study is included in the manuscript and the Supplementary Files.

## Author Contributions

FS, AG, and NvR study concept and design. FS and HE acquisition of data. FS and NvR analysis and interpretation of data. FS drafted the manuscript. FS, HE, PH, MS, AG, and NvR critical revision of the manuscript for important intellectual content. AG and NvR obtained the funding. PH, MS, AG, and NvR study supervision.

## Conflict of Interest Statement

The authors declare that the research was conducted in the absence of any commercial or financial relationships that could be construed as a potential conflict of interest.
