# Supplementary material for: In Silico Analysis Identifies Intestinal Transit as a Key Determinant of Systemic Bile Acid Metabolism
Source: Front Physiol. 2018 Jun 8;9:631. doi: 10.3389/fphys.2018.00631 (PMC6008656; doi:10.3389/fphys.2018.00631)
Supplement: Supplementary file 1 [file Data_Sheet_1.pdf]

Supplementary Material

In silico analysis identifies intestinal transit as a key determinant of systemic bile acid metabolism

Fianne L. P. Sips[1*], Hannah M. Eggink[2], Peter A. J. Hilbers[1], Maarten R. Soeters[2], Albert K. Groen[3,4], Natal A. W. van Riel[1,3]

*** Correspondence:** Fianne L.P. Sips: f.l.p.sips@tue.nl

1 Index of supplementary information

Supplementary Data 1. Model equations and implementation details (see section 2, p. 2).

Supplementary Data 2. Model Implementation "*BA model package*" (see separate zip-file).

Please read the accompanying README file for an overview of the functions and an introduction into model application.

Supplementary Tables (section 3, p. 15)

Supplementary Table 1. Model parameters (see section 3).

Supplementary Table 2. Model constants (see section 3).

Supplementary Table 3. Calibration dataset (see section 3).

Supplementary Table 4. Model validation (see section 3).

Supplementary Figures (section 4, p. 25)

Supplementary Figure 1. Calibration dataset.

Supplementary Figure 2. Simulated model outputs vs calibration dataset.

Supplementary Figure 3. Control of DCA pool by colonic and small intestinal transit speed**.**

Supplementary Figure 4. Cost function sensitivity and model identifiability.

Supplementary Figure 5. Spider plots of S_{PM} for all model parameters

Supplementary Figure 6. Investigation of the role of intestinal transit

Supplementary References (p. 34)

2 Supplementary Data1. Model equations and implementation details.

2.1 Mathematical model

The mathematical model represents the enterohepatic circulation of bile acids in humans as a series of connected compartments, governed by differential equations. In short, bile acids are produced in the liver compartment li, and transported to gallbladder (gb), small intestine (si_1 - si_{10}), colon (co_1 - co_5), and plasma (pl), or excreted via the feces. The bile acid pool in each compartment is in μmol, except for the plasma compartment, in which the bile acid concentration in $\mu mol/L$ is instead described. Transfer rates between compartments are generally given in $\mu mol/min$. The full model is described below per tissue; model parameters and constants are provided in Supplementary Tables 1 and 2.

2.1.1 Description and equations

Symbols

In the equations below, the symbol BA will denote any of [gCA, tCA, uCA, $gCDCA$, $tCDCA$, $uCDCA$, $gDCA$, $tDCA$, $uDCA$, $gUDCA$, $tUDCA$, $uUDCA$, $gLCA$, $tLCA$, $uLCA$, $gLCA_s$, $tLCA_s$, $uLCA_s$, gO, tO,uO]. cBA denotes conjugated bile acids, while unconjugated bile acids are denoted by uBA. Also, sulfated bile acids may be referred to as sBA, and the complementary group of non-sulfated bile acids as nBA.

Liver

In the liver, bile acids are synthesized, conjugated with taurine or glycine, taken up from both plasma and directly from the intestines via first pass clearance of portal blood, sulfated, and excreted towards gallbladder and small intestine. Bile acids are transported out of the hepatocyte by the Bile Salt Export Pump (BSEP) which has little or no affinity for unconjugated bile acids (Rossi et al., 1987), but does transport conjugated bile acids across the canalicular membrane. Therefore, bile acids are only transported out of the liver after (re-) conjugation. In the model, we assume for simplicity that all bile acids are conjugated as they appear in the liver, and thus by definition all bile acids in the liver are conjugated either with glycine or taurine. Since deconjugation will only take place in distal intestines, the model effectively does not contain unconjugated bile acids in liver, gallbladder, or the first compartments of the small intestine.

Bile acid ***synthesis*** of the species BA is denoted as ${r_u}^{cBA}$ ($\mu mol/min$) and depends on the total synthesis rate, as well as species- and conjugation status-dependent parameters. It is given by:

$$r_u^{cBA} = k_u \cdot \gamma_{BA} \cdot \gamma_{cs} \qquad \text{(S.1)}$$

Here, γ_{BA} is a unit less, species-dependent parameter that divides newly synthesized bile acids between the primary bile acids. Therefore, it should hold that all values of γ_{BA} are between 0 and 1 and $\sum \gamma_{BA} = 1$. Only CA and $CDCA$ are synthesized, so there are in fact two non-zero γ_{BA} in the model: γ_{CA} and γ_{CDCA} ($\gamma_{BA} = 0$ for $BA \in [DCA, UDCA, LCA, LCA_s, O]$). It must therefore hold that $\gamma_{CDCA} = 1 - \gamma_{CA}$.. Likewise, γ_{cs} are unit less parameters which divide newly synthesized BA between taurine- (γ_t) and glycine- (γ_g) conjugated BA. Again, γ_t and γ_g have a value between 0 and 1 and $\gamma_t + \gamma_g = 1$. Finally, k_u is a model parameter that represents the total synthesis of bile acids in $\mu mol/min$.

Input into the liver from ***first pass extraction*** - ${r_{li,in}}^{cBA}$ $(\mu mol/min)$ - is modelled as a direct input from the intestines. To implement the immediate re-conjugation of BA, first pass extraction for the unconjugated state is 0, and first pass extraction for glycine and taurine conjugated species are both a combination of the conjugated and unconjugated BA via:

$$ {r_{li,in}}^{cBA} = \psi^{cBA} \cdot {r_{x,si}}^{cBA} + \psi^{uBA} \cdot \gamma_{cs} \cdot \left({r_{x,si}}^{uBA} + {r_{x,co}}^{uBA} \right) \tag{S.2} $$

Again, the γ_{cs} divide the conjugated bile acids between g or t, and ${r_{x,si}}^{cBA}$, ${r_{x,si}}^{uBA}$ and ${r_{x,co}}^{uBA}$ are the fluxes of conjugated and unconjugated bile acids from the small intestine and colon, as described below. Note that we use x here to denote the fact that the flux does not have a single target compartment, but rather flows to both the liver and the plasma. ψ^{cBA} and ψ^{uBA} are the unitless hepatic extraction ratios for the conjugated and unconjugated BA, respectively – assumed to be constant over time (LaRusso et al., 1978). Since the affinity of the hepatic bile acid receptors has been shown to vary between bile acids (Dawson et al., 2009; Hofmann and Hagey, 2014; Suga et al., 2017), we do not assume homogeneous extraction of bile acids, but instead define different hepatic uptake parameters. We assume hepatic extraction is equal for the glycine and taurine conjugates of the same species, and that the bile acids can be divided into groups that are governed by similar kinetics. Therefore, we define separate hepatic extraction parameters for four different categories in the model: conjugated trihydroxylated bile acids (gCA and tCA) are governed by ψ_{tri}, conjugated dihydroxy bile acids (conjugates of $CDCA$, $UDCA$ and DCA) are governed by ψ_{di}, conjugated mono-hydroxylated bile acids (LCA and o) are governed by ψ_{mono} and conjugated sulfated species (LCA_s) are governed by ψ_s. The respective ψ^{uBA}, governing the extraction of the corresponding unconjugated groups, are calculated via

$$ \psi^{uBA} = \psi^{cBA} \cdot \psi_u \tag{S.3} $$

where ψ_u is again a unitless parameter between 0 and 1 which implements the less efficient extraction of uBA in comparison to cBA.

Sulfation of LCA takes place for both taurine and glycine conjugated BA via mass action kinetics as follows:

$$ r_{LCA_s,LCA} = k_{LCA_s,LCA} \cdot cLCA_{s_{li}} \tag{S.4} $$

This is the only transformation that takes place in the liver, and transformation can therefore be summarized as:

$$ \begin{aligned} {r_{li\,tr_{in}}}^{cBA} &= \begin{cases} r_{LCA_s,LCA} & for\ cLCA_s \\ 0 & otherwise \end{cases} \\ {r_{li\,tr_{out}}}^{cBA} &= \begin{cases} r_{LCA_s,LCA} & for\ cLCA \\ 0 & otherwise \end{cases} \end{aligned} \tag{S.5} $$

Similar to first pass extraction, bile acids are also extracted from the ***plasma*** pool to the glycine- or taurine- conjugated liver pools via

$$ {r_{li,pl}}^{cBA} = \psi^{cBA} \cdot Q_L \cdot [cBA_{pl}] + \psi^{uBA} \cdot \gamma_c \cdot Q_L \cdot [uBA_{pl}] \tag{S.6} $$

where Q_L (L/min) is the total plasma flow through the liver. Finally, BA are ***excreted from the liver*** via mass action kinetics governed by a parameter k_{xl} (where we again use x to reflect the fact that this flux will be split into gallbladder and duodenal components). Note that no unconjugated bile acids are excreted, and thus ${r_{x,li}}^{uBA} = 0$.

$$ {r_{x,li}}^{cBA} = k_{xl} \cdot cBA_{li} \qquad \text{(S.7)} $$

The final equation for (conjugated) bile acid kinetics in the liver is given in equation S.8.

$$ \frac{\mathrm{d}\, cBA_{li}}{\mathrm{d}\, t} = {r_u}^{cBA} + {r_{li,in}}^{cBA} + {r_{li,pl}}^{cBA} - {r_{x,li}}^{cBA} + {r_{li\,tr_{in}}}^{cBA} - {r_{li\,tr_{out}}}^{cBA} \qquad \text{(S.8)} $$

Gallbladder

After excretion from the liver, bile acids are either directly secreted into the first intestinal compartment si_1, or stored in gb. The kinetics of gallbladder bile acids are determined by a balance of input from the liver and output towards the duodenum. ***Influx from the liver*** is simply modelled as a fraction of liver output

$$ {r_{gb,li}}^{BA} = \gamma_{gb} \cdot {r_{x,li}}^{BA} , \qquad \text{(S.9)} $$

wherein γ_{gb} is again a parameter between 0 and 1. Note that the complementary flux to si_1 is now given by ${r_{si1,li}}^{BA} = (1 - \gamma_{gb}) \cdot {r_{x,li}}^{BA}$.

Outflux from the gallbladder, on the other hand, is meal dependent via

$$ {r_{si1,gb}}^{BA} = k_{xg} \cdot \rho\left((t-\tau), \beta_{gb}, \delta_{gb}\right) \cdot BA_{gb} \qquad \text{(S.10)} $$

Where k_{xg} is the fasting gallbladder emptying, and $\rho(t, \beta, \delta)$ is a normalized Rayleigh function which shows a transient increase with a peak location of β (min) and a postprandial increase of δ. In detail, $\rho\,(t, \beta, \delta) = 1 + \delta \cdot \left(\frac{t\sqrt{e}}{\beta}\, e^{-\frac{t^2}{2\cdot\beta^2}}\right)$.

The final equation for gallbladder kinetics is given in equation S.11:

$$ \frac{\mathrm{d}\, BA_{gb}}{\mathrm{d}\, t} = {r_{gb,li}}^{BA} - {r_{si1,gb}}^{BA} \qquad \text{(S.11)} $$

Small intestines

The intestines have been divided into 15 distinct compartments ($si1$ – $si10$ and $co1$ - $co5$) to form the bulk of the model. Starting from the first (duodenal) small intestine compartment si_1, bile acids are propelled through the consecutive compartments of the small intestine ($si_1, si_2, \ldots\ si_{10}$) and the compartments of the colon ($co_1, co_2, \ldots\ co_5$), and – if they are not taken up through the intestinal wall – excreted as a fecal output flux. Intestinal kinetics are dependent on time, conjugation status, sulfation status, and location in the intestines. Intestinal kinetics involve the following processes:

- Influx from gb and li
- Transit along consecutive compartments
- Deconjugation
- Transformation
- Passive uptake
- Active uptake
- Fecal excretion

Influx of BA from gallbladder and liver only occurs in compartment si_1. For simplicity, we will here define fluxes in the si and co over the entire intestines at once, and refer to $si_1 - si_{10}$ and $co_1 - co_5$ as $ic_1 - ic_{15}$. The influx of bile acids into the duodenum (S.12) is the result of the fluxes from liver and gallbladder:

$$r_{ic_n,x}{}^{BA} = \begin{cases} (1-\gamma_{gb}) \cdot \ r_{x,li}{}^{BA} + r_{si1,gb}{}^{BA} & if\ n = 1 \\ 0 & if\ n > 1 \end{cases} \tag{S.12}$$

Transit is modelled via a temporally and spatially heterogeneous transit parameter. Spatially, the intestines are divided into three transit regions ($si_1 - si_8$, $si_9 - si_{10}$, and $co_1 - co_5$), incorporating the decrease of transit speed along the small intestine (Quigley et al., 1984; Seidl et al., 2012), as well as the much slower passage through colon than small intestine (see CDS).

For the 15 consecutive intestinal compartments we define

$$r_{ic_{n+1},ic_n}{}^{BA} = k_{tr_{n+1,n}}(t) \cdot BA_{ic_n} \tag{S.13}$$

The input from transit ($r_{ic_n\,pr_{in}}{}^{BA}$) is thus given by $r_{ic_n,ic_{n-1}}{}^{BA}$, while the output from transit is $r_{ic_n\,pr_{out}}{}^{BA} = r_{ic_{n+1},ic_n}{}^{BA}$. The time-dependent transit parameters are based on one of three transit parameters ($k_{si,\alpha}$, $k_{si,\beta}$ or k_{co}) and display a transient response to meal as follows:

$$k_{tr_{n+1,n}}(t,\tau,\beta_{SI},\delta_{SI}) = \begin{cases} k_{si,\alpha} \cdot \rho\,((t-\tau),\beta_{SI},\delta_{SI}) & for\ n \in \{1,2,\ldots,8\} \\ k_{si,\beta} \cdot \rho\,((t-\tau),\beta_{SI},\delta_{SI}) & for\ n \in \{9,10\} \\ k_{co} \cdot \rho\,((t-\tau),\beta_{SI},\delta_{SI}) & for\ n \in \{11,12,\ldots,15\} \end{cases} \tag{S.14}$$

For the terminal compartment $n = 15$, the outflux is the flux leaving the body via ***fecal excretion***.

Deconjugation *in vivo* depends on the microbial content and concentration, and takes place in the terminal small intestine and colon. In the model, we divide the intestine into three zones with different microbial potential. Deconjugation in every intestinal compartment is given by

$$r_{d,ic_n}{}^{BA} = k_{uc} \cdot D_{bact,ic_n} \cdot BA_{ic_n}{}^{cBA}\ , \tag{S.15}$$

where deconjugation of taurine-conjugated BA takes place at a different rate to glycine-cojugated BA, and thus $k_{uc} = k_{ut}$ for taurine conjugated BA and $k_{uc} = k_{ug}$ for glycine-

conjugated BA. Furthermore, $D_{bact,ic_n} = \begin{cases} 0 & for\ n \in \{1,2,\dots,5\} \\ D_{bact,dsi} & for\ n \in \{6,7,\dots,10\} \\ 1 & for\ n \in \{11,12,\dots,15\} \end{cases}$, so that deconjugation does not take place in the proximal small intestine, and small intestinal deconjugation is normalized to deconjugation in the colon. Small intestinal deconjugation intensity is determined by parameter $D_{bact,dsi}$. These deconjugation fluxes are an influx for unconjugated BA and an outflux for conjugated BA as described in equation S.16.

$$r_{ic_n\,d_{in}}{}^{BA} = \begin{cases} r_{d,ic_n}{}^{gBA} + r_{d,ic_n}{}^{tBA} & for\ uBA \\ 0 & for\ cBA \end{cases}$$
$$r_{ic_n\,d_{out}}{}^{BA} = \begin{cases} 0 & for\ uBA \\ r_{d,ic_n}{}^{BA} & for\ cBA \end{cases} \quad (S.16)$$

Transformation of bile acids is only possible for unconjugated bile acids and takes place mainly in the colon. In the model, therefore, it is restricted to uBA in the colon compartments $co_1 - co_5$. The overview of possible transformations is given in the Main Text, and includes dehydroxylation of CA and CDCA to DCA and LCA respectively, and desulfation of LCA. For each transformation from BA_2 to BA_1 a parameter k_{BA_1,BA_2} is defined, such that

$$r_{BA_1,BA_2,co_n} = k_{BA_1,BA_2} \cdot uBA_{2_{co_n}} \quad (S.17)$$

Most transformations are set to 0, but nonzero parameters are defined for $k_{DCA,CA}$, $k_{UDCA,CDCA}$, $k_{LCA,CDCA}$, $k_{LCA,UDCA}$, desulfation via k_{LCA,LCA_s}, and transformation to other from any bile acid via $k_{oth,BA}$.

$$r_{ic_n\,tr_{in}}{}^{BA} = \begin{cases} \sum_{m=1}^{N_{BA}} r_{BA,BA_m,co_n} & for\ uBA \\ 0 & for\ cBA \end{cases}$$
$$r_{ic_n\,tr_{out}}{}^{BA} = \begin{cases} \sum_{m=1}^{N_{BA}} r_{BA_m,BA,co_n} & for\ uBA \\ 0 & for\ cBA \end{cases} \quad (S.18)$$

Passive uptake

Unconjugated – but not conjugated – bile acids may be taken up from the intestines via passive diffusion. For unconjugated bile acids, passive uptake is thus included in the model down the length of the intestines. However, since small intestinal compartments have different physical dimensions than colonic compartments, we define two different passive uptake rates:

$$r_{ic_n,PU}{}^{BA} = \begin{cases} k_{xi,pu,si} \cdot BA_{ic_n} & if\ BA \in uBA\ and\ n \in \{1,2,\dots,10\} \\ k_{xi,pu,co} \cdot BA_{ic_n} & if\ BA \in uBA\ and\ n \in \{11,12,\dots,15\} \\ 0 & if\ BA \in cBA \end{cases} \quad (S.19)$$

Active uptake

Active uptake is possible for both cBA and uBA, but – as the responsible transporter ASBT is localized only in the very end of the terminal ileum (Dawson et al., 2009; Dietschy, 1968; Håkansson et al., 2002; Kramer et al., 1993; Mottino et al., 2002; Stelzner et al., 2000b, 2000a), active uptake is only included in the final two compartments of the si. ASBT-mediated intestinal active uptake may be saturable (Krag and Phillips, 1974) and is therefore modelled via Michaelis-Menten kinetics. As sBA have a lower affinity for ASBT, a separate parameter $Vmax_s$ is defined to regulate sBA.

$$r_{ic_n,AU}{}^{BA} = \begin{cases} 0 & if\ n \in \{1, 2, \dots, 8, 11, 12, \dots, 15\} \\ \frac{V_{max,ss} \cdot BA_{ic_n}}{K_{m,ASBT} + \sum_{BA} \mathrm{BA}_{ic_n}} & if\ n \in \{9,10\} \end{cases} \tag{S.20}$$

Where $V_{max,ss} = V_{max}$ for $BA \in nBA$, and $V_{max,ss} = V_{max,s}$ for $BA \in sBA$.

In summary, the total intestinal kinetics are given by:

$$\frac{\mathrm{d}\, BA_{ic_n}}{\mathrm{d}\, t} = r_{ic_n,x}{}^{BA} + r_{ic_n\, pr_{in}}{}^{BA} - r_{ic_n\, pr_{out}}{}^{BA} + r_{ic_n\, d_{in}}{}^{BA} - r_{ic_n\, d_{out}}{}^{BA} + r_{ic_n\, tr_{in}}{}^{BA} - r_{ic_n\, tr_{out}}{}^{BA} - r_{ic_n,PU}{}^{BA} - r_{ic_n,AU}{}^{BA} \tag{S. 21}$$

Plasma

BA taken up from the intestines may be directly transported to the liver via first pass extraction (see equation S.2). The remainder of bile acids that have been taken up appear in circulating plasma, the dynamics of which are given below. Note that in the plasma bile acid levels are defined ($[\mathrm{BA}_{pl}]$) as a concentration.

$$\frac{\mathrm{d}\, [BA_{pl}]}{\mathrm{d}\, t} = \frac{r_{pl,in}{}^{BA} - r_{li,pl}{}^{BA}}{V_P} \tag{S.22}$$

Herein, V_P is the total plasma volume, $r_{li,pl}{}^{BA}$ can be derived from equation S.6 (note that in plasma, conjugated and unconjugated bile acids constitute separate fluxes), and $r_{pl,in}{}^{BA}$ is the inverse of the first pass extraction as given in equation S.2.

2.1.2 Additional calculations

Portal blood concentration

Portal concentrations of bile acids are not explicitly modelled, but are estimated based on the plasma concentrations and the direct flux from intestines to liver.

$$[cBA_{po}] = [cBA_{pl}] + \frac{r_{x,si}{}^{cBA}}{Q_P} \quad \text{(S.23)}$$

For the unconjugated bile acids, this is:

$$[uBA_{po}] = [uBA_{pl}{}^{u}] + \frac{r_{x,si}{}^{uBA} + r_{x,co}{}^{uBA}}{Q_P} \quad \text{(S.24)}$$

Intestinal transit time

One of the constraints used in developing the model is the intestinal transit time. To find the simulated transit times a simulation is run from an initial state in which all state variables are set to 0 apart from either si_1 or co_1 conjugated bile acid, which is set to 100. All dynamics and synthesis are then disabled, apart from transit of this pool of bile acids. The simulation is run either for a few hours without a meal (small intestinal transit, fasting), for a few hours with a meal at t=0 (small intestinal transit, feeding) or for several days, including multiple meals (colonic transit). The small intestinal or colonic pool is determined over the course of the simulation, and the transit time is defined as the time point at which 50 % of the initial pool of bile acids still remains in the tissue.

Fecal output

In order to track the cumulative bile acids excreted from the model, we include dummy states representing the feces.

$$\frac{\mathrm{d}\,BA_{fe}}{\mathrm{d}\,t} = +\,r_{\mathrm{ic}_{16},\mathrm{ic}_{15}}{}^{BA} \quad \text{(S.25)}$$

Pool

The whole body pool in the model is defined as

$$BA_{pool} = BA_{li} + BA_{gb} + BA_{pl} \cdot V_P + \sum_{n=1}^{10} BA_{si_n} + \sum_{m=1}^{5} BA_{co_m} \quad \text{(S.26)}$$

Fractional catabolic rates, finally, are calculated from pool size and synthesis rate (which equals catabolic rate in steady state).

2.2 Calibration dataset

To compose the CDS, data was gathered from the literature (Supplementary Table 3) describing bile acid composition, conjugation, sulfation, pool sizes, a number of fluxes, and the postprandial characteristics of bile acid metabolism, as well as intestinal transit times.

Data was only included if it could be directly related to a model variable. For instance, in evaluating biliary output measurements, data was only included if it was collected in a normal daily rhythm, and excluded if collected during long-term gallbladder stimulation. In the collection of composition data, particular attention is paid to whether the methods employed included sulfated bile acids – and in some tissues, separate compositions including and not including sulfated bile acids are composed.

From the collected data, a mean over all individuals was calculated. Whenever possible, mean composition, conjugation or sulfation was taken either directly from the text or calculated from individual measurements. In some cases, however, when only the mean absolute concentrations were available, an estimate of the mean composition, conjugation or sulfation was calculated, based on this data. Calculations were performed in Microsoft Excel (2010). In some cases, graphical data was digitized using PlotDigitizer (version 2.6.6).

In evaluation of the results of fitting the model to the dataset, it is important to realize that because different methods were used to collect the data, some biases or conflicts may be present in the dataset. Examples of this include the location of the peak of conjugated bile acids and total bile acids. Theoretically, these values should almost overlap, as the increase seen in total bile acids stems from an increase of conjugated bile acids. In practice, however, we observe a difference between the values which likely stems from the different experimental design (meal type, etc) used in the included sources. The mean cBA peak, finally, occurs at 118 minutes, whereas the total bile acid peak is observed at 95 minutes. The conjugated bile acid peak is thus 24 % later than the total bile acid peak. Similarly, calculating the percentage of the bile acid pool stored in the gallbladder from the absolute content and total pool size, and comparing this to the relative pool size in the gallbladder as calculated from individual body distributions also yield a difference of 26 %. In this case, we can presume the difference to – at least in part – stem from a correlation between total pool size and gallbladder pool content (Nilsell, 1990). Measurement uncertainty is not accounted for in the dataset, but based on these estimates of the conflicts within the dataset we estimate that 25 % is a typical value (see below). An overview of the CDS and the references to the data are provided in Supplementary Table 3.The data is visualized in Supplementary Figure 1.

2.3 Model simulation and optimization

2.3.1 Model simulation

To simulate the model to a dynamic steady state, we solve the differential equations from an empty initial state in two steps – first in fasting for n_f days, and then with meal dynamics for an additional n_d. Preliminary simulations showed that values of $n_f = 20$ and $n_d = 35$ gave good results – these values were chosen for all simulations.

The differential equations were solved with compiled MEX files, using the SUNDIALS CVode package (2.6.0, Lawrence Livermore National Laboratory, Livermore, California) (Hindmarsh et al., 2005; Vanlier et al., 2012), or with Matlab solver ode15s.

In addition to the basal model states, several states were included to calculate the cumulative flux through e.g. the duodenum, the ileocecal valve, and in fecal excretion.

2.3.2 Parameter optimization

Parameter optimization was performed by generating 10000 log-uniformly distributed random samples from the determined physiological range phr for each parameter (Supplementary Table 1). The normalized cost function value $nSSE$ was determined for each of these samples. The best 50 parameter sets were then chosen as an initial point for local optimization with local solver *lsqnonlin.* The best of these results was further refined locally. Weights w_i (see Main Text) for each datapoint are set to $\frac{1}{N}$ (where N is the number of species included in the composition).

2.4 Validation

2.4.1 Validation of parameter values against measured values

Estimates of parameter values of hepatic extraction were calculated from two sources: ψ_{tri}, ψ_{di}, ψ_{mono} and ψ_u were calculated from a group of healthy human subjects (Eggink et al., 2017), and ψ_u was also calculated from a group of healthy pigs (Eggink et al., 2017).

Human data

Following the determination of bile acid concentrations in portal and venous blood (Eggink et al., 2017), hepatic extraction (HE) for each bile acid was calculated assuming that (1) of the blood entering the liver, 75 % originates from the portal vein and (2) outside the portal / hepatic system, the concentration of bile acids in the blood in the fasting state is constant.

$$HE_{BA} = 1 - \frac{BA_V}{0.25 * BA_V + 0.75 * BA_P} \quad \text{(S.27)}$$

Here, HE_{BA} is the calculated hepatic extraction ratio for any bile acid BA, BA_V is the venous concentration of this BA ($\mu mol/L$), BA_P is the portal concentration of this BA ($\mu mol/L$). To

obtain an estimate of the parameter values, a mean is calculated over the available HE ratios of all BA that are governed by a parameter, and then over all subject. For example, for ψ_{tri}, the mean of the HE of gCA and tCA was calculated. Two subjects were excluded from the calculations, as the portal concentrations in these subjects were found to be lower than the venous concentrations, and the assumptions for the calculation of the hepatic extraction parameter do not hold in these circumstances.

Pig data

Porcine BA concentrations were determined during a mixed meal test in a transorgan flux model (Eggink et al., 2017; Schooneman et al., 2015) and included fasting state as well as postprandial plasma samples. This yielded individual bile acid concentrations at arterial (BA_A), portal (BA_P), and hepatic venous (BA_H) sampling sites, as well as portal (Q_P) and splanchic (Q_L) plasma flows. HE was then calculated via

$$HE_{BA} = 1 - \frac{BA_H \cdot Q_L}{BA_P \cdot Q_P + BA_A \cdot (Q_L - Q_P)} \tag{S.28}$$

The ratio of unconjugated to conjugated hepatic extraction was estimated based on the ratio of conjugated to unconjugated HE for CDCA and UDCA, which were calculated for each time point.

2.4.2 Independent variables and dynamics

Bile acid flux in duodenum, jejunum, ileum

For comparison of the intestinal bile acid fluxes with a measurement of such fluxes (LaRusso et al., 1978) we mimic the duodenal, jejunal and ileal sampling sites spanning the small intestine by looking at fluxes between the 2nd and 3rd 5th and 6th, and 8th and 9th compartments.

Cecal bile acid composition

To compare the model to measurements of cecal bile acid composition (Hamilton et al., 2007), we calculate the local composition of the bile acids in each colonic compartment. We compare this to the mean we calculate of the composition as determined by liquid chromatography – mass spectroscopy, and the composition as determined by gas chromatography – mass spectroscopy in (Hamilton et al., 2007).

Tracer kinetics

Kinetics of a Selenium-75-homocholic acid taurine ([75 Se] HCAT) bile acid tracer were simulated by including an additional tracer class of bile acids (tr) in the model. tr were prevented from undergoing any deconjugation in the intestine, and otherwise followed the kinetics of tCA. In the tracer kinetic simulation, a slightly different day rhythm was maintained, emulating the experimental procedure outlined in (Ferraris et al., 1992). The dose of tracer was introduced to the model at 5 PM, and subsequent sampling of the tracer was done by calculating the amount of tracer present in the gallbladder in the morning fasting state. The halflife was calculated based on these consecutive morning measurements. Similarly, to measure plasma tracer kinetics, conjugated tracer was introduced into pl.

2.4.3 *In silico* cholecystectomy

To simulate cholecystectomy, the gallbladder is taken out of play by setting the fraction of hepatic output that is sent to the gallbladder (γ_{gb}) to 0, rerouting all bile acids directly to the duodenal compartment si_1.

2.5 Sensitivity, identifiability and control

2.5.1 Sensitivity to plasma measures S_{PM}

To reiterate the Main Text, sensitivity of plasma measure $\boldsymbol{f}$ to a parameter p_k is given by:

$$S_{PM}{}^{\boldsymbol{f}}_{p_k} = \frac{1}{2\,\Delta\,N_f}\sum_{j=1}^{N_f}\frac{|f_j(p_k)-f_j(p_k+\Delta\cdot p_k)\,|+|f_j(p_k)-f_j(p_k-\Delta\cdot p_k)\,|}{f_j(p_k)} \tag{S.29}$$

This sensitivity is calculated for four (vector) functions representing plasma measures: total fasting bile acid concentration $f_{[BA_{pl}]_{total}}$, fasting bile acid composition $\boldsymbol{f_{composition}}$, fasting bile acid conjugation $\boldsymbol{f_{conjugation}}$ and the normalized postprandial response $\boldsymbol{f_{postprandial}}$. For simplicity, we display the composition of the 5 common, nonsulfated bile acids:

$$\boldsymbol{f_{composition}} = 100\ \cdot \frac{\begin{bmatrix}[CA_{pl}]\\ [CDCA_{pl}]\\ [DCA_{pl}]\\ [UDCA_{pl}]\\ [LCA_{pl}]\end{bmatrix}}{[CA_{pl}]+[CDCA_{pl}]+[DCA_{pl}]+[UDCA_{pl}]+[LCA_{pl}]} \tag{S.30}$$

The postprandial response is chosen as a vector of normalized total bile acid concentration concentrations at 0, 30, 60, 90, 120 and 150 minutes after a meal - chosen to mimic an experimental setup in which circulating bile acids are regularly measured.

2.5.2 Sensitivity to the sum of squared errors S_{nSSE}

To calculate the sensitivity to the cost function, we first define the squared error between the model and a single datapoint i as

$$E_i(\boldsymbol{p}) = \left(w_i\ \cdot\ \frac{y_i(\boldsymbol{p})-d_i}{d_i}\right)^2 \tag{S.31}$$

Notice that with this definition, the value of $nSSE(\boldsymbol{p})$ can be calculated as

$$nSSE(\boldsymbol{p}) = \sum_{i=1}^{N_{CDS}} E_i(\boldsymbol{p}) \tag{S.32}$$

We then calculate the sensitivity $S_{nSSE_{p_k}}$ with equation S.33.

$$S_{nSSE_{p_k}} = \frac{1}{2}\,\frac{\sum_{i=1}^{N_{CDS}}|E_i(p_k)-E_i(p_k+\Delta\cdot p_k)\,|+|E_i(p_k)-E_i(p_k-\Delta\cdot p_k)\,|}{\sum_{i=1}^{N_{CDS}} E_i(p_k)} \tag{S.33}$$

Note that in equation S.33 the $nSSE$ is summed before dividing it by its regular value, as residuals are centered around 0, and element-wise division would lead to overemphasis of residuals that are small in the optimal fit, leading to misleadingly high sensitivities.

2.5.3 Necessity of model components: simulation of other model variants

To investigate the amount of detail necessary in the description of intestinal transit, we proposed a total of four transit models – the previously described model in which both spatial and temporal heterogeneity is included being the most detailed.

In the most simple model ($M0$), small intestinal transit is governed by a single parameter $k_{si,\alpha}$, and intestinal transit is constant over time. This model was then expanded in one of two ways: (1) In MT, intestinal transit is transiently increased in response to a meal, propelling the intestinal contents forwards. (2) In MS, propulsion in the terminal ileum is governed by an additional parameter, allowing distal transit to be slower than transit in the proximal small intestine to facilitate absorption of e.g. bile acids. Now, two transit speeds are defined for the small intestine: one for the first 8 compartments ($k_{si,\alpha}$) and one for the final two compartments ($k_{si,\beta}$). Finally, the model in which both of these extensions have been combined (and for which the equations, simulation and optimization have been described above) is designated MST.

The $M0$, MS and MT models were implemented, simulated and optimized as described for the MST model. In each case the 10 000 sets of parameters were drawn from the $\boldsymbol{phr}$ of these parameters. Because MST is the most detailed model, the three alternative models have fewer parameters: $M0$ has 30 parameters, MT has 32, and MS has 31. The best 50 results were then reoptimized, yielding an optimal parameter set.

2.5.4 Profile likelihood analysis

To evaluate parameter identifiability, a profile likelihood analysis (Raue et al., 2009; Vanlier et al., 2013) was performed. The analysis involves stepwise increasing or decreasing the value of a single parameter away from the optimal value, and then reoptimizing all other (32) parameters freely, given the fixed value. This returns a profile of values of the $nSSE$ against the value of a parameter, and reveals for which values of a parameter the model is able to describe the data. We note that as we do not define standard deviations for our dataset, we cannot determine a confidence interval for our parameters – however, the result still provide information on the (un) identifiability of parameters.

2.5.5 Spider plots

Spider plots were generated for the components of S_{PM} and S_{nSSE}. For S_{PM}, the four individual sensitivities were simply combined in a radiating plot. For S_{nSSE}, the 76 components of the $nSSE$ were divided into the seven catagories presented in the Main Text (Figure 2): Composition, Conjugation, Sulphation, Pools sizes, Fluxes, Postprandial

Characteristics and Transit Times. The sensitivity $S_{nSSE,cat}$ is then calculated analougously to S_{nSSE} (equation S.33), by summing over the current category only.

2.5.6 Control plots

Control plots were generated to visualize the effect of changing a parameter on the plasma (and DCA) outcome measures. For the control plots, a single parameter was set to values between 1% and 105 % of its value, with steps of 1%. The high bound of 105 % was chosen such that no parameters are given a numeric value above their physiological bound.

2.6 Postprandial dynamics

To gain insight into the processes underlying the postprandial response, we simulated the model without these components. First, the model was initialized at a fasting state calculated with the regular parameter vector ($\boldsymbol{p_{normal}}$). From this point, the postprandial response of total bile acids ($R(t, \boldsymbol{p})$) is calculated with either (1) $\boldsymbol{p_{normal}}$, (2) with the postprandial increase in intestinal propulsion δ_{SI} set to 0 ($\boldsymbol{p_{\delta_{SI}=0}}$), or (3) with the liver outflux set to 0 ($\boldsymbol{p_{k_{xl}=0}}$). The contributions ($C(t)$) can then be calculated as follows:

$$C_{GI\ propulsion}(t) \quad = R(\boldsymbol{p}_{normal}) - R(\boldsymbol{p_{\delta_{SI}=0}}) \tag{S.34}$$

$$C_{Recycling}(t) \quad = R(\boldsymbol{p_{normal}}) - R(\boldsymbol{p_{k_{xl}=0}}) \tag{S.35}$$

$$C_{GB\ emptying}(t) \quad = R(\boldsymbol{p_{normal}}) - C_{GI\ propulsion}(t) - C_{Recycling}(t) - [BA]_{fasting} \tag{S.36}$$

3 Supplementary Tables

Supplementary Table 1. Model Parameters

**Symbol**	**Unit**	**Value**	**Bound ***	**Equation**
β_{GB}	min	70.3	[0.1 - 90]	S.10
δ_{GB}	$-$	44.3	[0.1 - 100]	S.10
β_{SI}	min	10.3	[0.1 - 60]	S.14
δ_{SI}	$-$	2.93	[0.1 - 100]	S.14
k_{xl}	$1/min$	0.118	[10^{-3} - 10]	S.7
γ_{GB}	$-$	0.948	[0.5 – 1]	S.9
k_{xg}	$1/min$	$2.87 \cdot 10^{-3}$	[4^{-4} - 0.04]	S.10
$k_{xi,up,si}$	$1/min$	$9.04 \cdot 10^{-5}$	[10^{-5} - 0.1]	S.19
$k_{xi,up,co}$	$1/min$	$1.92 \cdot 10^{-4}$	[10^{-5} - 0.1]	S.19
$V_{max,s}$	μmol $/min$	0.01	[0.01 - 100]	S.20
V_{max}	μmol $/min$	439	[0.1 - 10^3]	S.20
K_m	μmol	$9.66 \cdot 10^3$	[$1 - 10^4$]	S.20
ψ_u**	$-$	0.625	[0.625 – 1]	S.3
ψ_{tri}	$-$	0.952	[0.01 – 1]	S.2
ψ_{di}	$-$	0.839	[0.01 – 1]	S.2
ψ_{mono}	$-$	0.775	[0.01 – 1]	S.2

ψ_{sulf}	$-$	0.0435	[0.01 – 1]	S.2
γ_t	$-$	0.368	[0.01 - 1]	S.1
k_{ut}	$1/min$	$1.99 \cdot 10^{-2}$	[10^{-5} - 0.1]	S.15
k_{ug}	$1/min$	$2.52 \cdot 10^{-3}$	[10^{-5} - 0.1]	S.15
k_{bact}^{dsi}	$-$	$7.69 \cdot 10^{-2}$	[10^{-6} - 1]	S.15
$k_{si,\alpha}$	$1/min$	0.152	[$5 \cdot 10^{-3}$ – 0.5]	S.14
$k_{si,\beta}$	$1/min$	$1.04 \cdot 10^{-2}$	[$5 \cdot 10^{-3}$ – 0.5]	S.14
k_{co}	$1/min$	$2.12 \cdot 10^{-3}$	[$3 \cdot 10^{-4}$-$3 \cdot 10^{-2}$]	S.14
k_u	μmol $/min$	0.824	[0.1 - 10]	S.1
γ_{CA}	$-$	0.530	[0.2 - 1]	S.1
$k_{DCA,CA}$	$1/min$	$2.68 \cdot 10^{-3}$	[10^{-5} - 0.1]	S.17
$k_{UDCA,CDCA}$	$1/min$	$2.08 \cdot 10^{-3}$	[10^{-5} - 0.1]	S.17
$k_{LCA,CDCA}$	$1/min$	$1.73 \cdot 10^{-7}$	[10^{-7} - 0.1]	S.17
$k_{LCA,UDCA}$	$1/min$	$4.08 \cdot 10^{-3}$	[10^{-5} - 0.1]	S.17
$k_{LCA,LCAs}$	$1/min$	$7.26 \cdot 10^{-4}$	[10^{-6} - 0.01]	S.17
$k_{LCAs,LCA}$	$1/min$	$6.70 \cdot 10^{-2}$	[10^{-3} - 10]	S.17
$k_{oth,BA}$	$1/min$	$8.31 \cdot 10^{-5}$	[10^{-6} - 0.01]	S.17

* As initial parameter vectors are sampled from a lognormal distribution between bounds, lower bounds are chosen unequal to 0.

** Unconstrained optimizations yielded values of around 0.4 for parameter $\boldsymbol{\psi_u}$. This value was slightly lower than was determined in the literature (Ahlberg et al., 1977) (Angelin et al., 1982) (Einarsson et al., 1996) (Einarsson et al., 1985). Therefore, a lower bound of 0.625 was set based on this published data on the extraction of unconjugated bile acids

Supplementary Table 2. Model constants

Symbol	Unit	Value	Description
Q_L	$\frac{L}{min}$	0.825	Total hepatic plasma flow
Q_P	$\frac{L}{min}$	$\frac{3}{4} \cdot Q_L$	Portal plasma flow
V_P	L	3.15	Plasma volume (Adiels et al., 2005)

Supplementary Table 3. Overview of calibration dataset.

* Several previous studies were included in this publication.

Data type	Data name	Maximum # of studies	Maximum # of subjects	# of datapoints	References
Composition	Plasma composition (S+NS)	7	200	6	(Bathena et al., 2013; Bayerdörffer et al., 1995; De Giorgi et al., 2014; Humbert et al., 2012; Mannes et al., 1987; Murata et al., 1983; Stellaard and Paumgartner, 1987)
	Plasma composition (NS)	5	58	3	(Angelin et al., 1982; Angelin and Björkhem, 1977; Einarsson et al., 1985; Ewerth, 1982; Ewerth et al., 1985)
	Liver composition	4	36	5	(Akashi et al., 1983; Honda et al., 1995; Setchell et al., 1997; Yanagisawa et al., 1980)
	Synthesized composition	8	80	2	(Danzinger et al., 1973; Einarsson et al., 1974; Kurt Einarsson, 1985; McCormick et al., 1973; Nilsell et al., 1983; Stellaard et al., 1984; Vlahcevic et al., 1972a, 1972b)
	Bile composition (S+NS)	5	67	6	(Akashi et al., 1983; Dilger et al., 2012, 2014; Honda et al., 1995; Makino and Nakagawa, 1978; Rossi et al., 1987)
	Bile composition (NS)	7	158	6	(Fisher and Yousef, 1973; Fracchia et al., 1998, 1999; Kurt Einarsson, 1985; Nakayama and Nakagaki, 1980; Nilsell et al., 1983; Perwaiz et al., 2001; Reihnér et al., 1989)
	Portal composition	5	64	5	(Ahlberg et al., 1977; Einarsson et al., 1985, 1996; Ewerth et al., 1985; Reihnér et al., 1989)

Supplementary Table 3 (continued)

	Fecal composition	8	188	6	(Breuer et al., 1985, 1986; Humbert et al., 2012; Makino and Nakagawa, 1978; Reddy, 1981; Reddy et al., 1978; Reddy and Wynder, 1977; Tanida et al., 1981)
Conjugation	Plasma conjugation	5	242	3	(Bathena et al., 2013; De Giorgi et al., 2014; Humbert et al., 2012; Matysik et al., 2011; Scherer et al., 2009)
	Bile conjugation	4	41	2	(Dilger et al., 2012, 2014; Nakayama and Nakagaki, 1980; Perwaiz et al., 2001; Rossi et al., 1987)
	Fecal conjugation	4	74	2	(Breuer et al., 1985, 1986; Humbert et al., 2012; Tanida et al., 1981)
Sulfation	Plasma sulfation	5	211	2	(Bathena et al., 2013; De Giorgi et al., 2014; Humbert et al., 2012; Kato et al., 1996; Murata et al., 1983)
	Bile sulfation	3	47	1	(Fisher et al., 1991; Palmer and Bolt, 1971)
	Fecal sulfation	4	74	1	(Breuer et al., 1985, 1986; Humbert et al., 2012; Tanida et al., 1981)
Pool sizes	Plasmal pool	16	347	1	(Ahmad et al., 2013; Albaugh et al., 2015; Bathena et al., 2013; Bayerdörffer et al., 1995; De Barros et al., 1982; De Giorgi et al., 2014; Dirksen et al., 2013; Ewerth et al., 1985; Humbert et al., 2012; Lips et al., 2014; Mannes et al., 1987; Matysik et al., 2011; Patti et al., 2009; Sonne et al., 2013; Sørensen et al., 1981; Suzuki et al., 2014)
	Portal pool	5	64	1	(Ahlberg et al., 1977; Einarsson et al., 1985, 1996; Ewerth et al., 1985; Reihnér et al., 1989)

Supplementary Table 3 (continued)

	Liver pool	4	1569	1	(Akashi et al., 1983; Honda et al., 1995; Setchell et al., 1997; Yanagisawa et al., 1980) / (de la Grandmaison et al., 2001; Mathuramon et al., 2009; Molina and DiMaio, 2012, 2015)
	Gallbladder pool	19	974	2	(Fisher and Yousef, 1973; Ginanni Corradini et al., 1998; Keulemans et al., 1998; Nilsell, 1990; Perwaiz et al., 2001; Tamasawa et al., 1993) / (Bodegraven et al., 1998; Chan et al., 2004; Chapman et al., 1998; Donald et al., 1991; Everson et al., 1980; Gourtsoyiannis et al., 1995; Güliter et al., 2003; Jazrawi et al., 1983; Kishk et al., 1987; Luiking et al., 1998; Masclee et al., 1997; Nakeeb et al., 2006; Portincasa et al., 1995, 2000; Sari et al., 2003; Sengupta et al., 2006; Shoda et al., 1995; Van Erpecum et al., 1992; Veysey et al., 2001a) / (Jazrawi et al., 1983; Mok et al., 1980; Nilsell, 1990)
	Total pool	11	161	4	(Bennion et al., 1978; Danzinger et al., 1973; Einarsson et al., 1974; Jazrawi et al., 1983; Kurt Einarsson, 1985; McCormick et al., 1973; Mok et al., 1980; Nilsell, 1990; Nilsell et al., 1983; Northfield and Hofmann, 1975; Roda et al., 1978; Vlahcevic et al., 1972a, 1972b)
Fluxes	Synthesis	8	86	1	(Einarsson et al., 1974; Kurt Einarsson, 1985; McCormick et al., 1973; Nilsell et al., 1983; Northfield and Hofmann, 1975; Roda et al., 1978; Vlahcevic et al., 1972a, 1972b)
	Biliary output	3	24	2	(Mok et al., 1980; Northfield and Hofmann, 1975; Roda et al., 1978)

Supplementary Table 3 (continued)

	BA fractional catabolic rate	8	84	2	(Einarsson et al., 1974; Kurt Einarsson, 1985; McCormick et al., 1973; Nilsell et al., 1983; Northfield and Hofmann, 1975; Roda et al., 1978; Stellaard et al., 1984; Vlahcevic et al., 1972b, 1972a)
Postprandial	Maximal increase	9	148	3	(Ahmad et al., 2013; Angelin and Björkhem, 1977; De Barros et al., 1982; De Giorgi et al., 2014; Dirksen et al., 2013; Heuman et al., 1982; J. M. J. I. Salemans, 1993; LaRusso et al., 1978; Linnet, 1983; Sonne et al., 2013; Suzuki et al., 2014; Tobiasson et al., 1981)
	Rise at 30 minutes	5	120	3	(Angelin and Björkhem, 1977; De Barros et al., 1982; Heuman et al., 1982; J. M. J. I. Salemans, 1993; Linnet, 1983; Sonne et al., 2013; Suzuki et al., 2014; Tobiasson et al., 1981)
	Time of maximal value	7	143	3	(Ahmad et al., 2013; Angelin and Björkhem, 1977; De Barros et al., 1982; De Giorgi et al., 2014; Heuman et al., 1982; J. M. J. I. Salemans, 1993; LaRusso et al., 1978; Sonne et al., 2013; Suzuki et al., 2014; Tobiasson et al., 1981)
Transit	Fasting SI transit	1*	114	1	(Pišlar et al., 2015)
	Postprandial SI transit	1*	36	1	(Pišlar et al., 2015)
	Colon transit	9	405	1	(Arhan et al., 1981; Bouchoucha et al., 2006; Chan et al., 2004; Danquechin Dorval et al., 1994; Metcalf et al., 1987; Notghi et al., 1994; Pomerri et al., 2009; Santos et al., 2000; Yuan et al., 2012)
TOTAL		99	-	76	

Supplementary Table 4. Model validation.

Simulated values and parameters compared to experimental values for validation.

* Values calculated from individual data are given as mean ± SEM. Values obtained from literature are given as [range of reported values].

** Parameter has a large range (see Supplementary Figure 4)

*** Based on literature values of the ratio between hepatic extraction of unconjugated and conjugated bile acids, this parameter was bounded between 0.625-1

**** In Table S1, the literature data provided here for context was also used to constrain the range of ψ_u. The direct measurements given here were used solely as validation data, as can be seen by the fact that the value measured in human subjects is slightly below the range previously determined. However, the estimated parameter value of 0.625 corresponds well with the measurement of 0.59 ± 0.08.

Description	Simulation	Experimental data	
	Value	Type	Value*
**Parameter values**			
ψ_{tri}	0.95	Measurement, human	0.97 ± 0.005
		Literature (Gilmore and Thompson, 1981) (Marigold et al., 1982) (Berge-Henegouwen and Hofmann, 1983) (Linnet et al., 1984)	[0.8-0.9]
ψ_{di}	0.84	Measurement, human	0.82 ± 0.07
		Literature (Berge-Henegouwen and Hofmann, 1983) (Linnet et al., 1984)	[0.6-0.8]
ψ_{mono}	0.78**	Measurement, human	0.64 ± 0.08
		Literature (Einarsson et al., 1996)	0.78
ψ_u	0.625***	Measurement, human	0.59 ± 0.08****
		Measurement, pig	0.65 ± 0.02
		Literature (Ahlberg et al., 1977)' (Angelin et al., 1982)' (Einarsson et al., 1996)' (Einarsson et al., 1985)	[0.625-1]

Supplementary Table 4 (continued)

**Independent predictions**			
BA entering colon	4.5 % of secreted	Literature<br>(Ferrebee and Dawson, 2015; Lefebvre et al., 2009)	< 5 % of secreted
Colonic BA composition	Main Text, Figure 3B	Literature<br>(Hamilton et al., 2007)	-
Intestinal propulsion	Main Text, Figure 3C	Literature (LaRusso et al., 1978)	-
[75 Se] HCAT halflife[1]	1.67 days	Literature<br>(Ferraris et al., 1992)<br>(van Tilburg et al., 1991)<br>(Riemsma et al., 2013)	3.0 ± 0.2 days<br>> 2.8 days<br>2.6 days
Plasma tracer halflife	2.78 minutes	Literature (LaRusso et al., 1978)	2.03 minutes
**Simulation without gallbladder (Main Text, Figure 3D)**			
Fasting BO	Increased	Literature<br>(Berr et al., 1989; Malagelada et al., 1973; Nahrwold and Grossman, 1970; Peeters et al., 1980; Shaffer and Small, 1977; Sonne et al., 2013)	Increased
$[BA_{pl}]_{fasting}$	Increased	Literature<br>(Barrera et al., 2015; Roda et al., 1978; Schalm et al., 1978; Sonne et al., 2013)	Unchanged or slightly increased
BA pool	Decreased	Literature<br>(Almond et al., 1973; Berr et al., 1989; Hepner et al., 1974; Kullak-Ublick et al., 1995; Pomare and Heaton, 1973; Roda et al., 1978; Shaffer and Small, 1977)	Unchanged or decreased

[1] *In silico* [75 Se] HCAT halflife was found to be lower than the corresponding experimental value. We hypothesize that this can be explained by the absolute preclusion of deconjugation implemented in the model, whereas *in vivo* a small amount of bile acid may deconjugated (Jazrawi et al., 1988).

Supplementary Table 4 (continued)

% Secondary BA	Increased	Literature (Almond et al., 1973; Berr et al., 1989; Hepner et al., 1974; Kullak-Ublick et al., 1995; Malagelada et al., 1973; Pomare and Heaton, 1973; Roda et al., 1978; van der Linden et al., 1983)	Biliary secondary bile acids are unchanged or increased
Time of peak	Decreased	Literature (Roda et al., 1978; Schalm et al., 1978; Sonne et al., 2013)	Peak is seen to occur earlier
Height of peak	Decreased	Literature (Hofmann, 2009; LaRusso et al., 1974; Roda et al., 1978; Schalm et al., 1978; Sonne et al., 2013)	Increase is usually lower, higher in (Sonne et al., 2013)
FTR	Increased	Literature (Almond et al., 1973; Berr et al., 1989; Hepner et al., 1974; Kullak-Ublick et al., 1995; Pomare and Heaton, 1973; Roda et al., 1978)	The fractional transfer rate of CA to DCA is often increased

4 Supplementary Figures

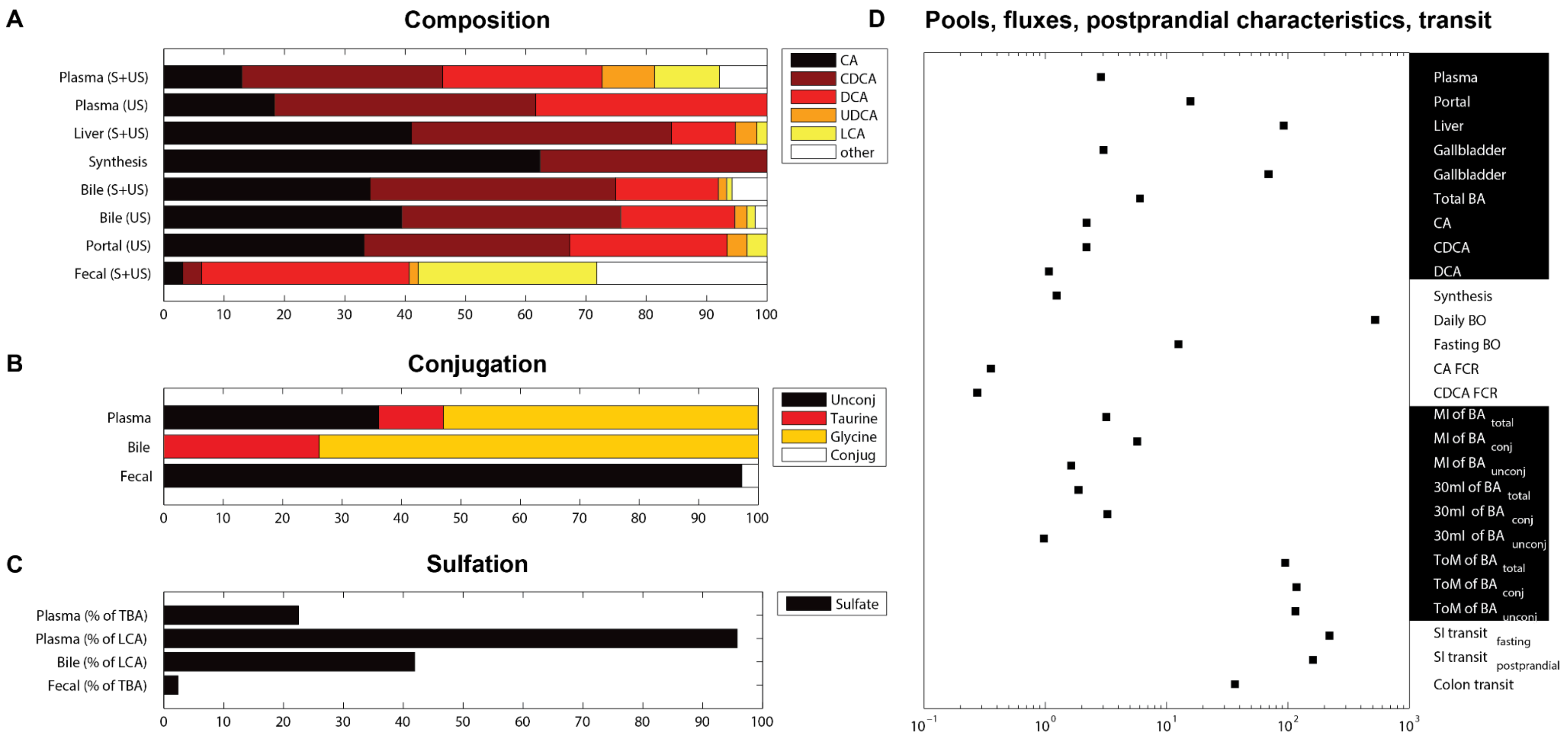

Supplementary Figure 1. (Previous page) Calibration dataset. (A) Composition data, describing the bile acid composition in plasma, liver, newly synthesized bile acids, bile, portal blood, and feces. For bile and plasma, compositions including and not-including sulfated bile acids are

simultaneously included. **(B)** The conjugation of bile acids in plasma, bile and feces. **(C)** Sulfation of LCA in bile and plasma, total sulfation in plasma and feces. **(D)** Organ (plasma, portal blood, liver, gallbladder) and bile acids species based (CA, CDCA, DCA, total) pools; fluxes (synthesis, physiological biliary output, and fractional catabolic rate); characteristics (30 minute increase $\boldsymbol{30mI}$, maximal increase $\boldsymbol{MI}$, and time of the maximal increase $\boldsymbol{ToM}$) of total bile acids, conjugated bile acids and unconjugated bile acids in peripheral blood in response to a meal; small intestinal transit after a meal and during fasting, and colonic transit

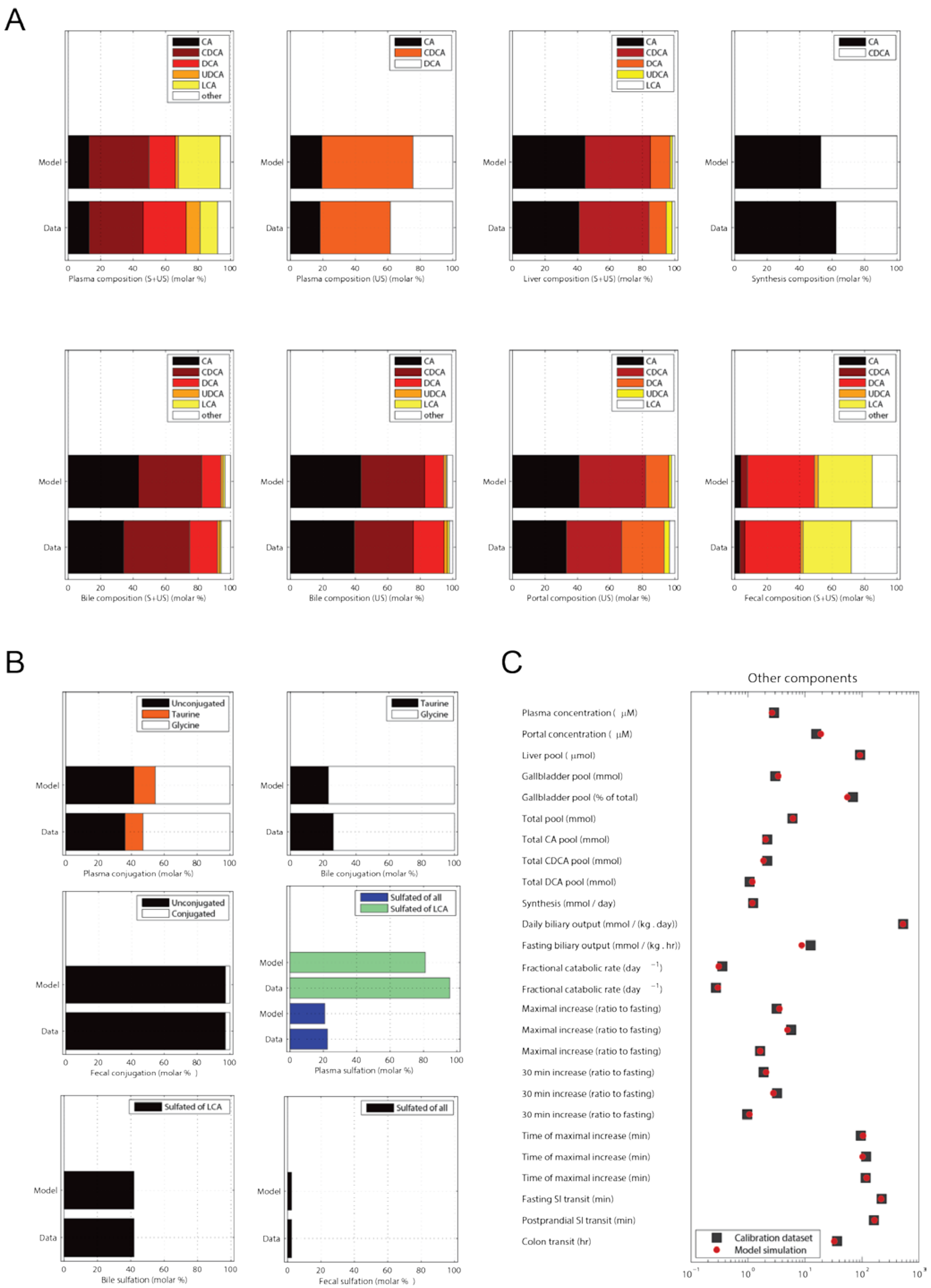
A
CA
CDCA
DCA
UDCA
LCA
other
Model
Data
Plasma composition (S+US) (molar %)
Plasma composition (US) (molar %)
Liver composition (S+US) (molar %)
Synthesis composition (molar %)
Bile composition (S+US) (molar %)
Bile composition (US) (molar %)
Portal composition (US) (molar %)
Fecal composition (S+US) (molar %)
B
Unconjugated
Taurine
Glycine
Conjugated
Plasma conjugation (molar %)
Bile conjugation (molar %)
Fecal conjugation (molar %)
Sulfated of all
Sulfated of LCA
Plasma sulfation (molar %)
Bile sulfation (molar %)
Fecal sulfation (molar %)
C
Other components
Plasma concentration (µM)
Portal concentration (µM)
Liver pool (µmol)
Gallbladder pool (mmol)
Gallbladder pool (% of total)
Total pool (mmol)
Total CA pool (mmol)
Total CDCA pool (mmol)
Total DCA pool (mmol)
Synthesis (mmol / day)
Daily biliary output (mmol / (kg . day))
Fasting biliary output (mmol / (kg . hr))
Fractional catabolic rate (day −1)
Fractional catabolic rate (day −1)
Maximal increase (ratio to fasting)
Maximal increase (ratio to fasting)
Maximal increase (ratio to fasting)
30 min increase (ratio to fasting)
30 min increase (ratio to fasting)
30 min increase (ratio to fasting)
Time of maximal increase (min)
Time of maximal increase (min)
Time of maximal increase (min)
Fasting SI transit (min)
Postprandial SI transit (min)
Colon transit (hr)
Calibration dataset
Model simulation

Supplementary Figure 2. (Previous page) Simulated model outputs vs calibration dataset. (A) Composition data. **(B)** Conjugation and sulfation data. **(C)** Absolute data (pool sizes, fluxes, postprandial characteristics, transit times).

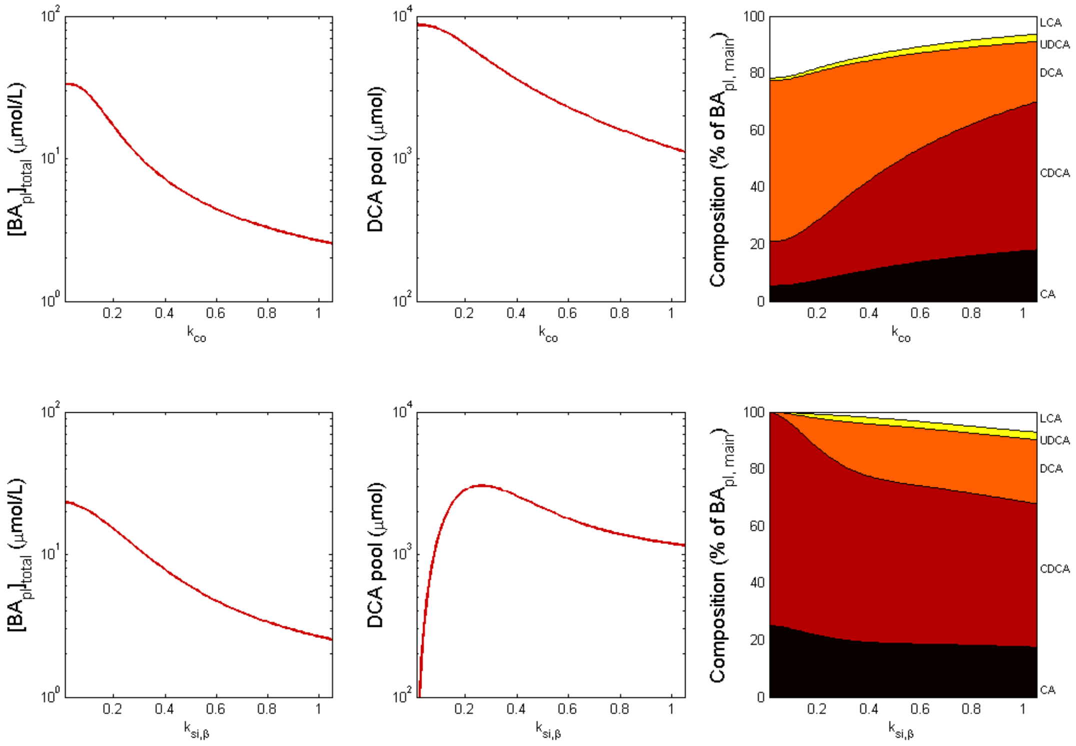

Supplementary Figure 3. Control of DCA pool by colonic and small intestinal transit speed. The top panel shows the control of $\boldsymbol{k_{co}}$ – i.e. the effect of changing the colon transit time on fasting plasma concentrations (left), DCA pool size (middle) and plasma composition (right). The bottom panel is as the top panel, but now for terminal small intestinal parameter $\boldsymbol{k_{si,\beta}}$. The control plots together show that when colonic transit slows down, the DCA pool and its contribution to the total bile acid pool increase. When small intestinal transit slows, on the other hand, the contribution of DCA decreases. This is in good agreement with the observations in (Marcus and Heaton, 1986) and **(Veysey et al., 2001b)**, which have previously revealed that colonic and small intestinal transit are important factors in determining DCA pool size. In (Marcus and Heaton, 1986), the DCA pool was found to decrease when colonic transit time was reduced with senna tablets, and conversely the DCA pool increasing when loperamide tablets were taken to increase colon residence time. Also, the effect of loperamide slowing down small intestinal pool and thus counteracting the effect of colonic slowing on the contribution is in agreement with the simulations, as the slowing of small intestinal transit via $\boldsymbol{k_{si,\beta}}$ also leads to a counteracting affect decreasing the DCA contribution. In accordance with **(Veysey et al., 2001b)**, slowing of only colonic transit time (in **(Veysey et al., 2001b)** with the aid of ocreotide) leads to an increased DCA pool, and an increase of the percentage of DCA in the serum composition.

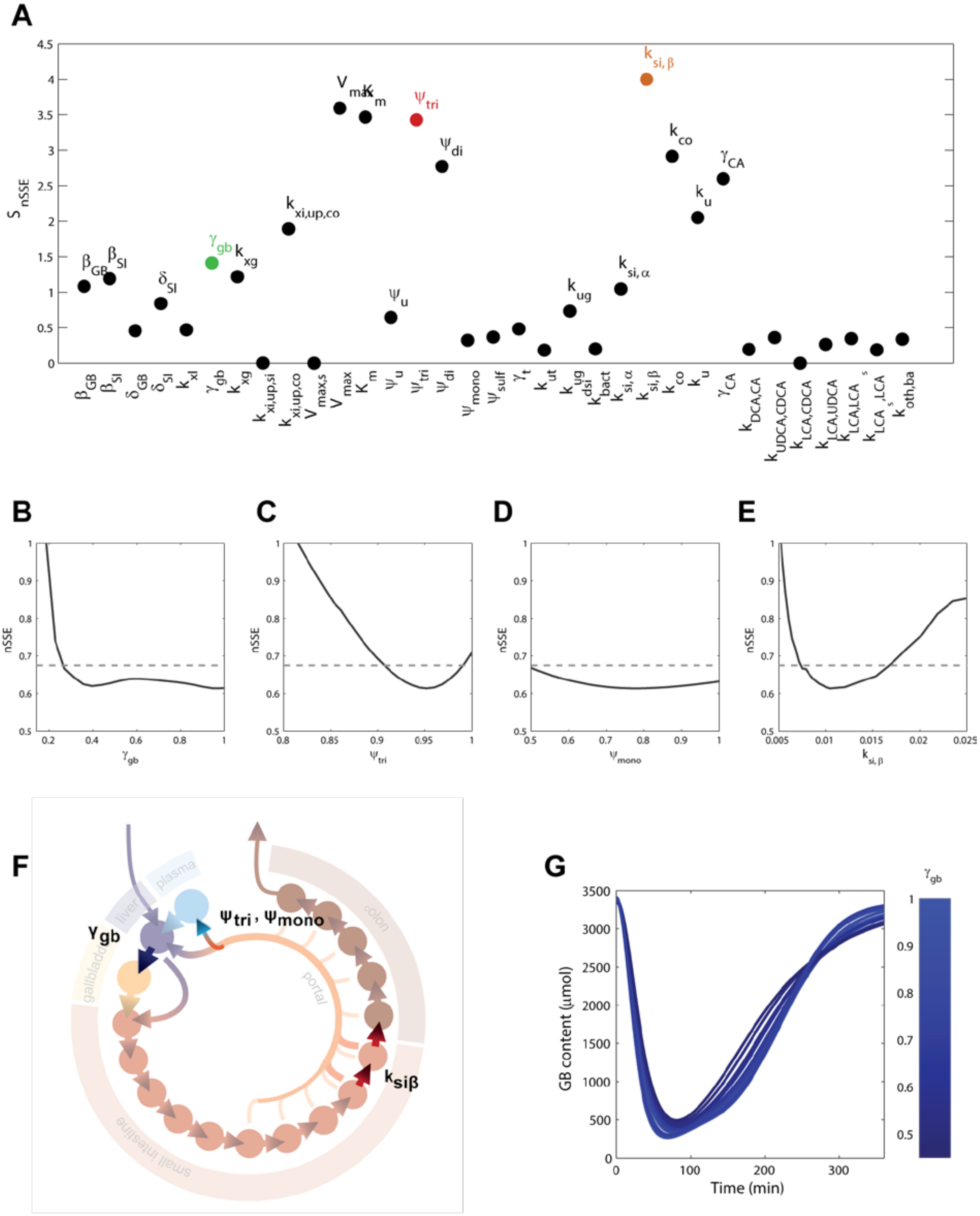

Supplementary Figure 4. Cost function sensitivity and model identifiability. (A) Sensitivity of the cost function to each of the 33 individual parameters. **(B)** Profile likelihood of fractional gallbladder entry parameter $\boldsymbol{\gamma_{gb}}$. **(C)** Profile likelihood of CA hepatic extraction parameter $\boldsymbol{\psi_{tri}}$. **(D)** Profile likelihood of hepatic extraction parameter $\boldsymbol{\psi_{mono}}$. **(E)** Profile likelihood of distal small intestinal transit parameter $\boldsymbol{k_{si,\beta}}$ **(F)** Schematic overview of the functionality of the parameter in B, C, D and E. **(G)** Gallbladder content profiles for values of $\boldsymbol{\gamma_{gb}}$ between 0.45-1 shows compensation in gallbladder kinetics by other parameters.

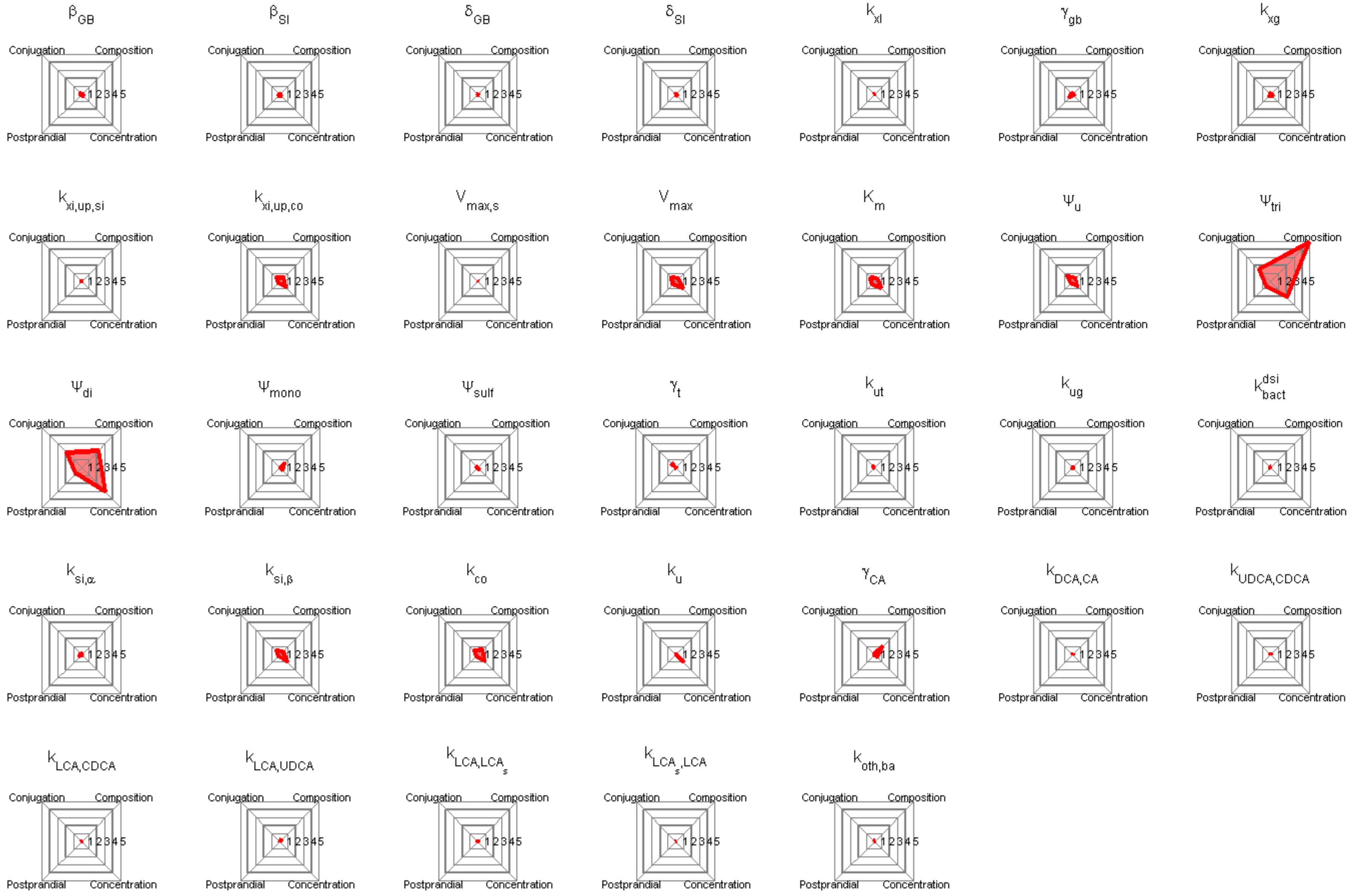

Supplementary Figure 5. Spider plots of S_{PM} for all model parameters

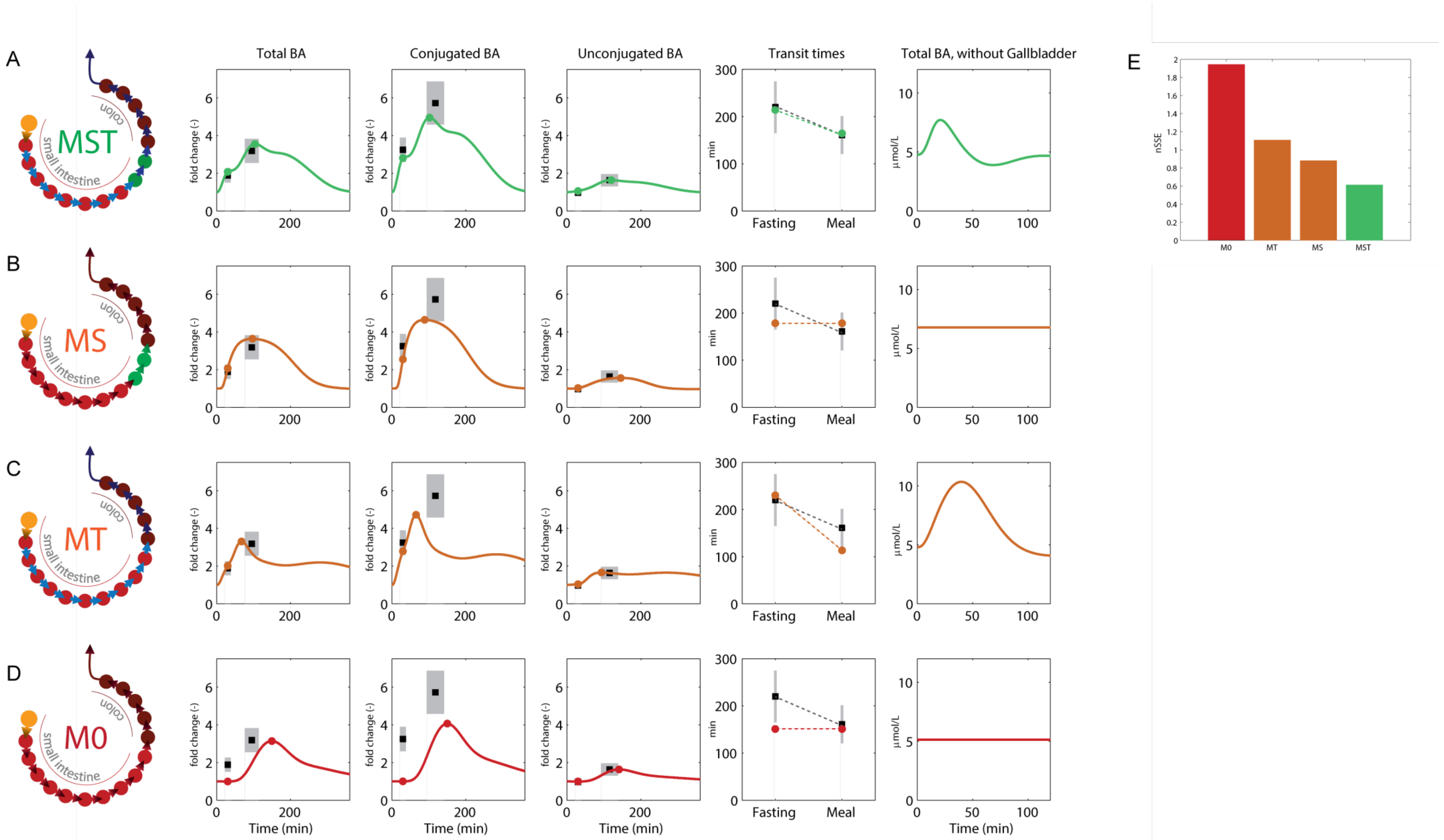

A
B
C
D
E
MST
MS
MT
M0
small intestine
colon
Total BA
Conjugated BA
Unconjugated BA
Transit times
Total BA, without Gallbladder
fold change (-)
min
µmol/L
Fasting
Meal
Time (min)
nSSE
M0
MT
MS
MST

Supplementary Figure 6. (Previous page) Investigation of the role of intestinal transit. (A-D) The best fit of the four models (M0, MT, MS and MST) are shown against the data for a subset of the calibration dataset. From left to right, the columns show (left) a visualization of the current model (2-4) the total, conjugated and unconjugated plasma meal responses, (5) the small intestinal transit data, and finally, in the right-most column, a qualitative evaluation of the plasma blood response after a meal following in silico cholecystectomy. For the postprandial responses, the precise location of the 30 minute point and peak are indicated by a square black marker, and the 25 % intervals for the 30 minute increase and peak increase (vertically) and location of the peak (horizontally) are visualized as grey boxes. For small intestinal transit, 25 % uncertainty is again visualized with grey boxes. In M0, small intestinal transit is homogeneous – described by a single parameter and not responsive to a meal. In MS, small intestinal transit is divided into a fast proximal section ($\boldsymbol{si_1}$-$\boldsymbol{si_8}$) and a slow second section ($\boldsymbol{si_9}$ and $\boldsymbol{si_{10}}$). In MT, intestinal transit is homogeneous over all sections as in M0, but after a meal, transit is transiently increased. In MST, both the temporal expansion of MT and the spatial expansion of MS are combined. **(E)** Values of *nSSE* for the optimal parameter set of each model.

5 Supplementary References

Adiels, M., Borén, J., Caslake, M. J., Stewart, P., Soro, A., Westerbacka, J., et al. (2005). Overproduction of VLDL1 Driven by Hyperglycemia Is a Dominant Feature of Diabetic Dyslipidemia. *Arterioscler. Thromb. Vasc. Biol.* 25, 1697–1703. doi:10.1161/01.ATV.0000172689.53992.25.

Ahlberg, J., Angelin, B., Björkhem, I., and Einarsson, K. (1977). Individual bile acids in portal venous and systemic blood serum of fasting man. *Gastroenterology* 73, 1377.

Ahmad, N., Pfalzer, A., and Kaplan, L. (2013). Roux-en-Y gastric bypass normalizes the blunted postprandial bile acid excursion associated with obesity. *Int. J. Obes. 2005* 37, 1553–1559. doi:10.1038/ijo.2013.38.

Akashi, Y., Miyazaki, H., and Nakayama, F. (1983). Correlation of bile acid composition between liver tissue and bile. *Clin. Chim. Acta Int. J. Clin. Chem.* 133, 125–132.

Albaugh, V. L., Flynn, C. R., Cai, S., Xiao, Y., Tamboli, R. A., and Abumrad, N. N. (2015). Early Increases in Bile Acids Post Roux-en-Y Gastric Bypass Are Driven by Insulin-Sensitizing, Secondary Bile Acids. *J. Clin. Endocrinol. Metab.* 100, E1225–E1233. doi:10.1210/jc.2015-2467.

Almond, H. R., Vlahcevic, Z. R., Bell, C. C. J., Gregory, D. H., and Swell, L. (1973). Bile Acid Pools, Kinetics and Biliary Lipid Composition before and after Cholecystectomy. *N. Engl. J. Med.* 289, 1213–1216. doi:10.1056/NEJM197312062892302.

Angelin, B., and Björkhem, I. (1977). Postprandial serum bile acids in healthy man. Evidence for differences in absorptive pattern between individual bile acids. *Gut* 18, 606–609.

Angelin, B., Björkhem, I., Einarsson, K., and Ewerth, S. (1982). Hepatic Uptake of Bile Acids in Man. *J. Clin. Invest.* 70, 724–731.

Arhan, P., Devroede, G., Jehannin, B., Lanza, M., Faverdin, C., Dornic, C., et al. (1981). Segmental colonic transit time. *Dis. Colon Rectum* 24, 625–629. doi:10.1007/BF02605761.

Barrera, F., Azócar, L., Molina, H., Schalper, K. A., Ocares, M., Liberona, J., et al. (2015). Effect of cholecystectomy on bile acid synthesis and circulating levels of fibroblast growth factor 19. *ResearchGate* 14, 710–21.

Bathena, S. P. R., Mukherjee, S., Olivera, M., and Alnouti, Y. (2013). The profile of bile acids and their sulfate metabolites in human urine and serum. *J. Chromatogr. B* 942–943, 53–62. doi:10.1016/j.jchromb.2013.10.019.

Bayerdörffer, E., Mannes, G. A., Ochsenkühn, T., Dirschedl, P., Wiebecke, B., and Paumgartner, G. (1995). Unconjugated secondary bile acids in the serum of patients with colorectal adenomas. *Gut* 36, 268–273.

Bennion, L. J., Drobny, E., Knowler, W. C., Ginsberg, R. L., Garnick, M. B., Adler, R. D., et al. (1978). Sex differences in the size of bile acid pools. *Metabolism.* 27, 961–969.

Berge-Henegouwen, G. P. V., and Hofmann, A. F. (1983). Systemic spill-over of bile acids. *Eur. J. Clin. Invest.* 13, 433–437. doi:10.1111/j.1365-2362.1983.tb00125.x.

Berr, F., Stellaard, F., Pratschke, E., and Paumgartner, G. (1989). Effects of cholecystectomy on the kinetics of primary and secondary bile acids. *J. Clin. Invest.* 83, 1541–1550.

Bodegraven, A. A. V., Böhmer, C. J., Manoliu, R. A., Paalman, E., Klis, A. H. V. der, Roex, A. J., et al. (1998). Gallbladder contents and fasting gallbladder volumes during and after pregnancy. *Scand. J. Gastroenterol.* 33, 993–7.

Bouchoucha, M., Devroede, G., Faye, A., Toumelin, P. L., Arhan, P., and Arsac, M. (2006). Colonic response to food in constipation. *Int. J. Colorectal Dis.* 21, 826–833. doi:10.1007/s00384-005-0787-5.

Breuer, D. N. F., Jaekel, S., Dommes, P., and Goebell, H. (1986). Fecal bile acid excretion pattern in cholecystectomized patients. *Dig. Dis. Sci.* 31, 953–960. doi:10.1007/BF01303216.

Breuer, N. F., Dommes, P., Jaekel, S., and Goebell, H. (1985). Fecal bile acid excretion pattern in colonic cancer patients. *Dig. Dis. Sci.* 30, 852–859.

Chan, D.-C., Chang, T.-M., Chen, C.-J., Chen, T.-W., Yu, J.-C., and Liu, Y.-C. (2004). Gallbladder contractility and volume characteristics in gallstone dyspepsia. *World J. Gastroenterol.* 10, 721–724. doi:10.3748/wjg.v10.i5.721.

Chan, Y.-K., Kwan, A. C.-P., Yuen, H., Yeung, Y.-W., Lai, K.-C., Wu, J., et al. (2004). Normal colon transit time in healthy Chinese adults in Hong Kong. *J. Gastroenterol. Hepatol.* 19, 1270–1275.

Chapman, B. A., Chapman, T. M., Frampton, C. M., Chisholm, R. J., Allan, R. B., Wilson, I. R., et al. (1998). Gallbladder Volume (Comparison of Diabetics and Controls). *Dig. Dis. Sci.* 43, 344–348. doi:10.1023/A:1018810523399.

Danquechin Dorval, E., Barbieux, J. P., Picon, L., Alison, D., Codjovi, P., and Rouleau, P. (1994). [Simplified measurement of colonic transit time by one radiography of the abdomen and a single type of marker. Normal values in 82 volunteers related to the sexes]. *Gastroenterol. Clin. Biol.* 18, 141–144.

Danzinger, R. G., Hofmann, A. F., Thistle, J. L., and Schoenfield, L. J. (1973). Effect of Oral Chenodeoxycholic Acid on Bile Acid Kinetics and Biliary Lipid Composition in Women with Cholelithiasis. *J. Clin. Invest.* 52, 2809–2821.

Dawson, P. A., Lan, T., and Rao, A. (2009). Bile acid transporters. *J. Lipid Res.* 50, 2340–2357. doi:10.1194/jlr.R900012-JLR200.

De Barros, S. G., Balistreri, W. F., Soloway, R. D., Weiss, S. G., Miller, P. C., and Soper, K. (1982). Response of total and individual serum bile acids to endogenous and exogenous bile acid

input to the enterohepatic circulation. *Gastroenterology* 82, 647–652. doi:10.5555/uri:pii:0016508582903067.

De Giorgi, S., Campos, V., Egli, L., Toepel, U., Carrel, G., Cariou, B., et al. (2014). Long-term effects of Roux-en-Y gastric bypass on postprandial plasma lipid and bile acids kinetics in female non diabetic subjects: A cross-sectional pilot study. *Clin. Nutr. Edinb. Scotl.* doi:10.1016/j.clnu.2014.09.018.

de la Grandmaison, G. L., Clairand, I., and Durigon, M. (2001). Organ weight in 684 adult autopsies: new tables for a Caucasoid population. *Forensic Sci. Int.* 119, 149–154. doi:10.1016/S0379-0738(00)00401-1.

Dietschy, J. M. (1968). Mechanisms for the intestinal absorption of bile acids. *J. Lipid Res.* 9, 297–309.

Dilger, K., Hohenester, S., Winkler-Budenhofer, U., Bastiaansen, B. A. J., Schaap, F. G., Rust, C., et al. (2012). Effect of ursodeoxycholic acid on bile acid profiles and intestinal detoxification machinery in primary biliary cirrhosis and health. *J. Hepatol.* 57, 133–140. doi:10.1016/j.jhep.2012.02.014.

Dilger, K., Hohenester, S., Winkler-Budenhofer, U., Bastiaansen, B. A. J., Schaap, F. G., Rust, C., et al. (2014). Corrigendum to: “Effect of ursodeoxycholic acid on bile acid profiles and intestinal detoxification machinery in primary biliary cirrhosis and health” [J Hepatol 2012;57:133–140]. *J. Hepatol.* 60, 684. doi:10.1016/j.jhep.2013.11.027.

Dirksen, C., Jørgensen, N. B., Bojsen-Møller, K. N., Kielgast, U., Jacobsen, S. H., Clausen, T. R., et al. (2013). Gut hormones, early dumping and resting energy expenditure in patients with good and poor weight loss response after Roux-en-Y gastric bypass. *Int. J. Obes.* 37, 1452–1459. doi:10.1038/ijo.2013.15.

Donald, J. J., Fache, J. S., Buckley, A. R., and Burhenne, H. J. (1991). Gallbladder contractility: variation in normal subjects. *Am. J. Roentgenol.* 157, 753–756. doi:10.2214/ajr.157.4.1892030.

Eggink, H. M., van Nierop, F. S., Schooneman, M. G., Boelen, A., Kalsbeek, A., Koehorst, M., et al. (2017). Transhepatic bile acid kinetics in pigs and humans. *Clin. Nutr.* doi:10.1016/j.clnu.2017.06.015.

Einarsson, K., Ahlberg, J., Angelin, B., Björkhem, I., and Ewerth, S. (1985). Portal venous bile acids in cholesterol gallstone disease: Effect of treatment with chenodeoxycholic and cholic acids. *Hepatology* 5, 661–665. doi:10.1002/hep.1840050423.

Einarsson, K., Alvelius, G., Hillebrant, C.-G., Reihnér, E., and Björkhem, I. (1996). Concentration of unsulfated lithocholic acid in portal and systemic venous plasma: evidence that lithocholic acid does not down regulate the hepatic cholesterol 7α-hydroxylase activity in gallstone patients. *Biochim. Biophys. Acta BBA - Mol. Basis Dis.* 1317, 19–26. doi:10.1016/0925-4439(96)00030-0.

Einarsson, K., Hellström, K., and Kallner, M. (1974). Bile Acid Kinetics in Relation to Sex, Serum Lipids, Body Weights, and Gallbladder Disease in Patients with Various Types of Hyperlipoproteinemia. *J. Clin. Invest.* 54, 1301–1311.

Everson, G. T., Braverman, D. Z., Johnson, M. L., and Kern, F. (1980). A critical evaluation of real-time ultrasonography for the study of gallbladder volume and contraction. *Gastroenterology* 79, 40–46. doi:10.5555/uri:pii:0016508580900724.

Ewerth, S. (1982). Postprandial serum concentration of individual bile acids in man. Influence of ileal resection. *Scand. J. Gastroenterol.* 17, 781–784.

Ewerth, S., Angelin, B., Einarsson, K., Nilsell, K., and Björkhem, I. (1985). Serum concentrations of ursodeoxycholic acid in portal venous and systemic venous blood of fasting humans as determined by isotope dilution-mass spectrometry. *Gastroenterology* 88, 126–133.

Ferraris, R., Galatola, G., Barlotta, A., Pellerito, R., Fracchia, M., Cottino, F., et al. (1992). Measurement of bile acid half-life using [75Se]HCAT in health and intestinal diseases. *Dig. Dis. Sci.* 37, 225–232. doi:10.1007/BF01308176.

Ferrebee, C. B., and Dawson, P. A. (2015). Metabolic effects of intestinal absorption and enterohepatic cycling of bile acids. *Acta Pharm. Sin. B* 5, 129–134. doi:10.1016/j.apsb.2015.01.001.

Fisher, M. M., and Yousef, I. M. (1973). Sex differences in the bile acid composition of human bile: Studies in patients with and without gallstones. *Can. Med. Assoc. J.* 109, 190–193.

Fisher, R. L., Hofmann, A. F., Converse, J. L., Rossi, S. S., and Lan, S. P. (1991). The lack of relationship between hepatotoxicity and lithocholic-acid sulfation in biliary bile acids during chenodiol therapy in the National Cooperative Gallstone Study. *Hepatol. Baltim. Md* 14, 454–463.

Fracchia, M., Pellegrino, S., Secreto, P., Calgaro, M., Taraglio, S., Pera, A., et al. (1999). Biliary bile acid composition in gastric cancer. *Int. J. Clin. Lab. Res.* 29, 46–48. doi:10.1007/s005990050062.

Fracchia, M., Pellegrino, S., Secreto, P., Pera, A., and Galatola, G. (1998). Biliary lipid composition in idiopathic bile acid malabsorption. *Gut* 43, 812–816.

Gilmore, I. T., and Thompson, R. P. H. (1981). Direct Measurement of Hepatic Extraction of Bile Acids in Subjects with and without Liver Disease. *Clin. Sci.* 60, 65–72. doi:10.1042/cs0600065.

Ginanni Corradini, S., Yamashita, G., Nuutinen, H., Chernosky, A., Williams, C., Hays, L., et al. (1998). Human gallbladder mucosal function: effects on intraluminal fluid and lipid composition in health and disease. *Dig. Dis. Sci.* 43, 335–343.

Gourtsoyiannis, N. C., Damilakis, J. E., Charoulakis, N. Z., Bakantaki, A. S., Vlahonikolis, J. G., and Xynos, E. (1995). Relationship of gallbladder contour, fasting volume and emptying to body size indices in normal subjects and patients with gallstones. *Digestion* 56, 395–399.

Güliter, S., Yilmaz, S., and Karakan, T. (2003). Evaluation of gallbladder volume and motility in non-insulin-dependent diabetes mellitus patients using real-time ultrasonography. *J. Clin. Gastroenterol.* 37, 288–291.

Håkansson, P., Andersson, I., Nyström, S., Löfgren, L., Amrot, L. F., and Li, H. (2002). Ontogenetic development and spatial distribution of the ileal apical sodium-dependent bile acid transporter and the ileal lipid-binding protein in apoE knockout and C57BL/6 mice. *Scand. J. Gastroenterol.* 37, 1089–1096.

Hamilton, J. P., Xie, G., Raufman, J.-P., Hogan, S., Griffin, T. L., Packard, C. A., et al. (2007). Human cecal bile acids: concentration and spectrum. *Am. J. Physiol. - Gastrointest. Liver Physiol.* 293, G256–G263. doi:10.1152/ajpgi.00027.2007.

Hepner, G. W., Hofmann, A. F., Malagelada, J. R., Szczepanik, P. A., and Klein, P. D. (1974). Increased Bacterial Degradation of Bile Acids in Cholecystectomized Patients. *Gastroenterology* 66, 556–564. doi:10.1016/S0016-5085(74)80044-2.

Heuman, R., Sjödahl, R., Tobiasson, P., and Tagesson, C. (1982). Postprandial serum bile acids in resected and non-resected patients with Crohn's disease. *Scand. J. Gastroenterol.* 17, 137–140.

Hindmarsh, A. C., Brown, P. N., Grant, K. E., Lee, S. L., Serban, R., Shumaker, D. E., et al. (2005). SUNDIALS: Suite of Nonlinear and Differential/Algebraic Equation Solvers. *ACM Trans Math Softw* 31, 363–396. doi:10.1145/1089014.1089020.

Hofmann, A. F. (2009). Bile acids: Trying to understand their chemistry and biology with the hope of helping patients. *Hepatology* 49, 1403–1418. doi:10.1002/hep.22789.

Hofmann, A. F., and Hagey, L. R. (2014). Key discoveries in bile acid chemistry and biology and their clinical applications: history of the last eight decades. *J. Lipid Res.* 55, 1553–1595. doi:10.1194/jlr.R049437.

Honda, A., Yoshida, T., Tanaka, N., Matsuzaki, Y., He, B., Shoda, J., et al. (1995). Increased bile acid concentration in liver tissue with cholesterol gallstone disease. *J. Gastroenterol.* 30, 61–66. doi:10.1007/BF01211376.

Humbert, L., Maubert, M. A., Wolf, C., Duboc, H., Mahé, M., Farabos, D., et al. (2012). Bile acid profiling in human biological samples: Comparison of extraction procedures and application to normal and cholestatic patients. *J. Chromatogr. B* 899, 135–145. doi:10.1016/j.jchromb.2012.05.015.

J. M. J. I. Salemans, F. M. N. (1993). Effect of ageing on postprandial conjugated and unconjugated serum bile acid levels in healthy subjects. *Eur. J. Clin. Invest.* 23, 192–8. doi:10.1111/j.1365-2362.1993.tb00761.x.

Jazrawi, R. P., Ferraris, R., Bridges, C., and Northfield, T. C. (1988). Kinetics for the synthetic bile acid 75selenohomocholic acid-taurine in humans: comparison with [14C]taurocholate. *Gastroenterology* 95, 164–169.

Jazrawi, R. P., Kupfer, R. M., Bridges, C., Joseph, A., and Northfield, T. C. (1983). Assessment of Gall-Bladder Storage Function in Man. *Clin. Sci.* 65, 185–191. doi:10.1042/cs0650185.

Kato, T., Yoneda, M., Nakamura, K., and Makino, I. (1996). Enzymatic determination of serum 3 alpha-sulfated bile acids concentration with bile acid 3 alpha-sulfate sulfohydrolase. *Dig. Dis. Sci.* 41, 1564–1570.

Keulemans, Y. C., Mok, K. S., de Wit, L. T., Gouma, D. J., and Groen, A. K. (1998). Hepatic bile versus gallbladder bile: A comparison of protein and lipid concentration and composition in cholesterol gallstone patients. *Hepatology* 28, 11–16. doi:10.1002/hep.510280103.

Kishk, S., Darweesh, R., Dodds, W., Lawson, T., Stewart, E., Kern, M., et al. (1987). Sonographic evaluation of resting gallbladder volume and postprandial emptying in patients with gallstones. *Am. J. Roentgenol.* 148, 875–879. doi:10.2214/ajr.148.5.875.

Krag, E., and Phillips, S. F. (1974). Active and Passive Bile Acid Absorption in Man. PERFUSION STUDIES OF THE ILEUM AND JEJUNUM. *J. Clin. Invest.* 53, 1686–1694.

Kramer, W., Girbig, F., Gutjahr, U., Kowalewski, S., Jouvenal, K., Müller, G., et al. (1993). Intestinal bile acid absorption. Na(+)-dependent bile acid transport activity in rabbit small intestine correlates with the coexpression of an integral 93-kDa and a peripheral 14-kDa bile acid-binding membrane protein along the duodenum-ileum axis. *J. Biol. Chem.* 268, 18035–18046.

Kullak-Ublick, G.-A., Paumgartner, G., and Berr, F. (1995). Long-term effects of cholecystectomy on bile acid metabolism. *Hepatology* 21, 41–45. doi:10.1002/hep.1840210109.

Kurt Einarsson, K. N. (1985). Influence of Age on Secretion of Cholesterol and Synthesis of Bile Acids by the Liver. *N. Engl. J. Med.* 313, 277–82. doi:10.1056/NEJM198508013130501.

LaRusso, N. F., Hoffman, N. E., Korman, M. G., Hofmann, D. A. F., and Cowen, A. E. (1978). Determinants of fasting and postprandial serum bile acid levels in healthy man. *Am. J. Dig. Dis.* 23, 385–391. doi:10.1007/BF01072919.

LaRusso, N. F., Korman, M. G., Hoffman, N. E., and Hofmann, A. F. (1974). Dynamics of the enterohepatic circulation of bile acids. Postprandial serum concentrations of conjugates of cholic acid in health, cholecystectomized patients, and patients with bile acid malabsorption. *N. Engl. J. Med.* 291, 689–692. doi:10.1056/NEJM197410032911401.

Lefebvre, P., Cariou, B., Lien, F., Kuipers, F., and Staels, B. (2009). Role of Bile Acids and Bile Acid Receptors in Metabolic Regulation. *Physiol. Rev.* 89, 147–191. doi:10.1152/physrev.00010.2008.

Linnet, K. (1983). Postprandial plasma concentrations of glycine and taurine conjugated bile acids in healthy subjects. *Gut* 24, 249–252.

Linnet, K., Andersen, J. R., and Hesselfeldt, P. (1984). Concentrations of glycine- and taurine-conjugated bile acids in portal and systemic venous serum in man. *Scand. J. Gastroenterol.* 19, 575–578.

Lips, M. A., de Groot, G. H., Berends, F. J., Wiezer, R., van Wagensveld, B. A., Swank, D. J., et al. (2014). Calorie restriction and Roux-en-Y gastric bypass have opposing effects on circulating FGF21 in morbidly obese subjects. *Clin. Endocrinol. (Oxf.)* 81, 862–870. doi:10.1111/cen.12496.

Luiking, Y. C., Peeters, T. L., Stolk, M. F. J., Nieuwenhuijs, V. B., Portincasa, P., Depoortere, I., et al. (1998). Motilin induces gall bladder emptying and antral contractions in the fasted state in humans. *Gut* 42, 830–835. doi:10.1136/gut.42.6.830.

Makino, I., and Nakagawa, S. (1978). Changes in biliary lipid and biliary bile acid composition in patients after administration of ursodeoxycholic acid. *J. Lipid Res.* 19, 723–728.

Malagelada, J. R., Go, V. L. W., Summerskill, W. H. J., and Gamble, W. S. (1973). Bile acid secretion and biliary bile acid composition altered by cholecystectomy. *Am. J. Dig. Dis.* 18, 455–459. doi:10.1007/BF01076595.

Mannes, G. A., Stellaard, F., and Paumgartner, G. (1987). Diagnostic sensitivity of fasting and postprandial serum bile acids determined by different methods. *Clin. Chim. Acta* 162, 147–154. doi:10.1016/0009-8981(87)90446-3.

Marcus, S. N., and Heaton, K. W. (1986). Intestinal transit, deoxycholic acid and the cholesterol saturation of bile--three inter-related factors. *Gut* 27, 550–558.

Marigold, J. H., Bull, H. J., Gilmore, I. T., Coltart, D. J., and Thompson, R. P. (1982). Direct measurement of hepatic extraction of chenodeoxycholic acid and ursodeoxycholic acid in man. *Clin. Sci. Lond. Engl. 1979* 63, 197–203.

Masclee, A. A., Gielkens, H. A., Lieverse, R. J., Penning, C., Schipper, J., and Lamers, C. B. (1997). Gallbladder motility in response to sham feeding and cholecystokinin in lean and obese subjects. *Digestion* 58, 43–49.

Mathuramon, P., Chirachariyavej, T., Peonim, A. V. M. V., and Rochanawutanon, M. (2009). Correlation of internal organ weight with body weight and length in normal Thai adults. *J. Med. Assoc. Thail. Chotmaihet Thangphaet* 92, 250–258.

Matysik, S., Martin, J., Bala, M., Scherer, M., Schäffler, A., and Schmitz, G. (2011). Bile acid signaling after an oral glucose tolerance test. *Chem. Phys. Lipids* 164, 525–529. doi:10.1016/j.chemphyslip.2011.05.003.

McCormick, W. C., Bell, C. C., Swell, L., and Vlahcevic, Z. R. (1973). Cholic acid synthesis as an index of the severity of liver disease in man. *Gut* 14, 895–902. doi:10.1136/gut.14.11.895.

Metcalf, A. M., Phillips, S. F., Zinsmeister, A. R., MacCarty, R. L., Beart, R. W., and Wolff, B. G. (1987). Simplified assessment of segmental colonic transit. *Gastroenterology* 92, 40–47.

Mok, H. Y. I., Bergmann, K. V., and Grundy, S. M. (1980). Kinetics of the enterohepatic circulation during fasting: Biliary lipid secretion and gallbladder storage. *Gastroenterology* 78, 1023–1033. doi:10.5555/uri:pii:0016508580907878.

Molina, D. K., and DiMaio, V. J. M. (2012). Normal organ weights in men: part II-the brain, lungs, liver, spleen, and kidneys. *Am. J. Forensic Med. Pathol.* 33, 368–372. doi:10.1097/PAF.0b013e31823d29ad.

Molina, D. K., and DiMaio, V. J. M. (2015). Normal Organ Weights in Women: Part II-The Brain, Lungs, Liver, Spleen, and Kidneys. *Am. J. Forensic Med. Pathol.* 36, 182–187. doi:10.1097/PAF.0000000000000175.

Mottino, A. D., Hoffman, T., Dawson, P. A., Luquita, M. G., Monti, J. A., Pozzi, E. J. S., et al. (2002). Increased expression of ileal apical sodium-dependent bile acid transporter in postpartum rats. *Am. J. Physiol. - Gastrointest. Liver Physiol.* 282, G41–G50. doi:10.1152/ajpgi.00309.2001.

Murata, N., Beppu, T., Takikawa, H., Otsuka, H., Kasama, T., and Seyama, Y. (1983). Determination of sulfated and nonsulfated bile acids in serum by mass fragmentography. *Steroids* 42, 575–592. doi:10.1016/0039-128X(83)90121-6.

Nahrwold, D. L., and Grossman, M. I. (1970). Effect of cholecystectomy on bile flow and composition in response to food. *Am. J. Surg.* 119, 30–34.

Nakayama, F., and Nakagaki, M. (1980). Quantitative determination of bile acids in bile with reversed-phase high-performance liquid chromatography. *J. Chromatogr.* 183, 287–293.

Nakeeb, A., Comuzzie, A. G., Al-Azzawi, H., Sonnenberg, G. E., Kissebah, A. H., and Pitt, H. A. (2006). Insulin resistance causes human gallbladder dysmotility. *J. Gastrointest. Surg. Off. J. Soc. Surg. Aliment. Tract* 10, 940–948; discussion 948-949. doi:10.1016/j.gassur.2006.04.005.

Nilsell, K. (1990). Bile acid pool size and gallbladder storage capacity in gallstone disease. *Scand. J. Gastroenterol.* 25, 389–394.

Nilsell, K., Angelin, B., Leijd, B., and Einarsson, K. (1983). Comparative Effects of Ursodeoxycholic Acid and Chenodeoxycholic Acid on Bile Acid Kinetics and Biliary Lipid Secretion in Humans. *Gastroenterology* 85, 1248–1256. doi:10.1016/S0016-5085(83)80003-1.

Northfield, T. C., and Hofmann, A. F. (1975). Biliary lipid output during three meals and an overnight fast. I. Relationship to bile acid pool size and cholesterol saturation of bile in gallstone and control subjects. *Gut* 16, 1–11.

Notghi, A., Hutchinson, R., Kumar, D., Smith, N. B., and Harding, L. K. (1994). Simplified method for the measurement of segmental colonic transit time. *Gut* 35, 976–981.

Palmer, R. H., and Bolt, M. G. (1971). Bile acid sulfates. I. Synthesis of lithocholic acid sulfates and their identification in human bile. *J. Lipid Res.* 12, 671–679.

Patti, M.-E., Houten, S. M., Bianco, A. C., Bernier, R., Larsen, P. R., Holst, J. J., et al. (2009). Serum bile acids are higher in humans with prior gastric bypass: potential contribution to improved glucose and lipid metabolism. *Obes. Silver Spring Md* 17, 1671–1677. doi:10.1038/oby.2009.102.

Peeters, T. L., Vantrappen, G., and Janssens, J. (1980). Bile acid output and the interdigestive migrating motor complex in normals and in cholecystectomy patients. *Gastroenterology* 79, 678–681.

Perwaiz, S., Tuchweber, B., Mignault, D., Gilat, T., and Yousef, I. M. (2001). Determination of bile acids in biological fluids by liquid chromatography-electrospray tandem mass spectrometry. *J. Lipid Res.* 42, 114–119.

Pišlar, M., Brelih, H., Mrhar, A., and Bogataj, M. (2015). Analysis of small intestinal transit and colon arrival times of non-disintegrating tablets administered in the fasted state. *Eur. J. Pharm. Sci.* 75, 131–141. doi:10.1016/j.ejps.2015.03.001.

Pomare, E. W., and Heaton, K. W. (1973). The effect of cholecystectomy on bile salt metabolism. *Gut* 14, 753–762.

Pomerri, F., Dodi, G., Nardin, M., and Muzzio, P. C. (2009). Colonic total and segmental transit times in healthy Italian adults. *Radiol. Med. (Torino)* 114, 925–934. doi:10.1007/s11547-009-0409-3.

Portincasa, P., Altomare, D. F., Moschetta, A., Baldassarre, G., Di Ciaula, A., Venneman, N. G., et al. (2000). The effect of acute oral erythromycin on gallbladder motility and on upper gastrointestinal symptoms in gastrectomized patients with and without gallstones: a randomized, placebo-controlled ultrasonographic study. *Am. J. Gastroenterol.* 95, 3444–3451. doi:10.1111/j.1572-0241.2000.03282.x.

Portincasa, P., Di Ciaula, A., Palmieri, V., Van Berge-Henegouwen, G. P., and Palasciano, G. (1995). Effects of cholestyramine on gallbladder and gastric emptying in obese and lean subjects. *Eur. J. Clin. Invest.* 25, 746–753.

Quigley, E. M., Borody, T. J., Phillips, S. F., Wienbeck, M., Tucker, R. L., and Haddad, A. (1984). Motility of the terminal ileum and ileocecal sphincter in healthy humans. *Gastroenterology* 87, 857–866.

Raue, A., Kreutz, C., Maiwald, T., Bachmann, J., Schilling, M., Klingmüller, U., et al. (2009). Structural and practical identifiability analysis of partially observed dynamical models by exploiting the profile likelihood. *Bioinforma. Oxf. Engl.* 25, 1923–1929. doi:10.1093/bioinformatics/btp358.

Reddy, B. S. (1981). Diet and Excretion of Bile Acids. *Cancer Res.* 41, 3766–3768.

Reddy, B. S., Hedges, A. R., Laakso, K., and Wynder, E. L. (1978). Metabolic epidemiology of large bowel cancer: fecal bulk and constituents of high-risk North American and low-risk Finnish population. *Cancer* 42, 2832–2838.

Reddy, B. S., and Wynder, E. L. (1977). Metabolic epidemiology of colon cancer: Fecal bile acids and neutral sterols in colon cancer patients and patients with adenomatous polyps. *Cancer* 39, 2533–2539. doi:10.1002/1097-0142(197706)39:6<2533::AID-CNCR2820390634>3.0.CO;2-X.

Reihnér, E., Björkhem, I., Angelin, B., Ewerth, S., and Einarsson, K. (1989). Bile acid synthesis in humans: regulation of hepatic microsomal cholesterol 7 alpha-hydroxylase activity. *Gastroenterology* 97, 1498–1505.

Riemsma, R., Al, M., Ramos, I. C., Deshpande, S. N., Armstrong, N., Lee, Y.-C., et al. (2013). *Background and definition of the decision problem(s)*. NIHR Journals Library Available at: https://www.ncbi.nlm.nih.gov/books/NBK262922/ [Accessed November 4, 2016].

Roda, E., Aldini, R., Mazzella, G., Roda, A., Sama, C., Festi, D., et al. (1978). Enterohepatic circulation of bile acids after cholecystectomy. *Gut* 19, 640–649. doi:10.1136/gut.19.7.640.

Rossi, S. S., Converse, J. L., and Hofmann, A. F. (1987). High pressure liquid chromatographic analysis of conjugated bile acids in human bile: simultaneous resolution of sulfated and unsulfated lithocholyl amidates and the common conjugated bile acids. *J. Lipid Res.* 28, 589–595.

Santos, S. L., Barcelos, I. K., and Mesquita, M. A. (2000). Total and segmental colonic transit time in constipated patients with Chagas□ disease without megaesophagus or megacolon. *Braz. J. Med. Biol. Res.* 33, 43–49. doi:10.1590/S0100-879X2000000100006.

Sari, R., Balci, M. K., Coban, E., and Karayalcin, U. (2003). Sonographic evaluation of gallbladder volume and ejection fraction in obese women without gallstones. *J. Clin. Ultrasound* 31, 352–357. doi:10.1002/jcu.10191.

Schalm, S. W., Larusso, N. F., Hofmann, A. F., Hoffman, N. E., Van Berge-Henegouwen, G. P., and Korman, M. G. (1978). Diurnal serum levels of primary conjugated bile acids. *Gut* 19, 1006–1014.

Scherer, M., Gnewuch, C., Schmitz, G., and Liebisch, G. (2009). Rapid quantification of bile acids and their conjugates in serum by liquid chromatography–tandem mass spectrometry. *J. Chromatogr. B* 877, 3920–3925. doi:10.1016/j.jchromb.2009.09.038.

Schooneman, M. G., Have, G. A. M. T., Vlies, N. van, Houten, S. M., Deutz, N. E. P., and Soeters, M. R. (2015). Transorgan fluxes in a porcine model reveal a central role for liver in acylcarnitine metabolism. *Am. J. Physiol. - Endocrinol. Metab.* 309, E256–E264. doi:10.1152/ajpendo.00503.2014.

Seidl, H., Gundling, F., Pfeiffer, A., Pehl, C., Schepp, W., and Schmidt, T. (2012). Comparison of small-bowel motility of the human jejunum and ileum. *Neurogastroenterol. Motil.* 24, e373–e380. doi:10.1111/j.1365-2982.2012.01955.x.

Sengupta, S., Modak, P., McCAULEY, N., and O'donnell, L. J. D. (2006). Effect of oral clarithromycin on gall-bladder motility in normal subjects and those with gall-stones. *Aliment. Pharmacol. Ther.* 24, 95–99. doi:10.1111/j.1365-2036.2006.02962.x.

Setchell, K., Rodrigues, C., Clerici, C., Solinas, A., Morelli, A., Gartung, C., et al. (1997). Bile acid concentrations in human and rat liver tissue and in hepatocyte nuclei. *Gastroenterology* 112, 226–235. doi:10.1016/S0016-5085(97)70239-7.

Shaffer, E. A., and Small, D. M. (1977). Biliary lipid secretion in cholesterol gallstone disease. The effect of cholecystectomy and obesity. *J. Clin. Invest.* 59, 828–840. doi:10.1172/JCI108705.

Shoda, J., He, B.-F., Tanaka, N., Matsuzaki, Y., Osuga, T., Yamamori, S., et al. (1995). Increase of deoxycholate in supersaturated bile of patients with cholesterol gallstone disease and its correlation with de novo syntheses of cholesterol and bile acids in liver, gallbladder emptying, and small intestinal transit. *Hepatology* 21, 1291–1302. doi:10.1002/hep.1840210512.

Sonne, D. P., Hare, K. J., Martens, P., Rehfeld, J. F., Holst, J. J., Vilsbøll, T., et al. (2013). Postprandial gut hormone responses and glucose metabolism in cholecystectomized patients. *Am. J. Physiol. - Gastrointest. Liver Physiol.* 304, G413–G419. doi:10.1152/ajpgi.00435.2012.

Sørensen, T. I., Bruusgaard, A., Nyboe Andersen, A., and Andersen, B. (1981). Serum levels and clearance of bile acids are unaffected by jejunoileal bypass with 3:1 or 1:3 jejunoileal ratio. *Scand. J. Gastroenterol.* 16, 705–711.

Stellaard, F., and Paumgartner, G. (1987). Quantitation of serum bile acids by isotope dilution with 13C-labelled homologs. *Clin. Chim. Acta* 162, 45–51. doi:10.1016/0009-8981(87)90231-2.

Stellaard, F., Sackmann, M., Sauerbruch, T., and Paumgartner, G. (1984). Simultaneous determination of cholic acid and chenodeoxycholic acid pool sizes and fractional turnover rates in human serum using 13C-labeled bile acids. *J. Lipid Res.* 25, 1313–1319.

Stelzner, M., Hoagland, V., and Somasundaram, S. (2000a). Distribution of bile acid absorption and bile acid transporter gene message in the hamster ileum. *Pflüg. Arch. Eur. J. Physiol.* 440, 157–162.

Stelzner, M., Somasundaram, S., and Kearney, D. J. (2000b). Distribution of bile acid transport capacities in the human ileum. *Gastroenterology* 118, A77. doi:10.1016/S0016-5085(00)82384-7.

Suga, T., Yamaguchi, H., Sato, T., Maekawa, M., Goto, J., and Mano, N. (2017). Preference of Conjugated Bile Acids over Unconjugated Bile Acids as Substrates for OATP1B1 and OATP1B3. *PLoS ONE* 12. doi:10.1371/journal.pone.0169719.

Suzuki, T., Aoyama, J., Hashimoto, M., Ohara, M., Futami-Suda, S., Suzuki, K., et al. (2014). Correlation between postprandial bile acids and body fat mass in healthy normal-weight subjects. *Clin. Biochem.* 47, 1128–1131. doi:10.1016/j.clinbiochem.2014.04.025.

Tamasawa, N., Yoneda, M., Makino, I., Takebe, K., Sone, K., and Kogawa, R. (1993). The effect of biliary bile acid concentration and composition on the calcium level in human gallbladder bile. *Tohoku J. Exp. Med.* 171, 297–307.

Tanida, N., Hikasa, Y., Hosomi, M., Satomi, M., Oohama, I., and Shimoyama, T. (1981). Fecal bile acid analysis in healthy japanese subjects using a lipophilic anion exchanger, capillary column gas chromatography and mass spectrometry. *Gastroenterol. Jpn.* 16, 363–371. doi:10.1007/BF02774469.

Tobiasson, P., Frydén, A., and Tagesson, C. (1981). Serum Bile Acids after Test Meals and Oral Load of Chenodeoxycholic Acid. *Scand. J. Gastroenterol.* 16, 763–767. doi:10.3109/00365528109181001.

van der Linden, W., Katzenstein, B., and Nakayama, F. (1983). The possible carcinogenic effect of cholecystectomy. No postoperative increase in the proportion of secondary bile acids. *Cancer* 52, 1265–1268. doi:10.1002/1097-0142(19831001)52:7<1265::AID-CNCR2820520722>3.0.CO;2-8.

Van Erpecum, K. J., Van Berge Henegouwen, G. P., Stolk, M. F. J., Hopman, W. P. M., Jansen, J. B. M. J., and Lamers, C. B. H. W. (1992). Fasting gallbladder volume, postprandial emptying and cholecystokinin release in gallstone patients and normal subjects. *J. Hepatol.* 14, 194–202. doi:10.1016/0168-8278(92)90158-L.

van Tilburg, A. J., de Rooij, F. W., van den Berg, J. W., Kooij, P. P., and van Blankenstein, M. (1991). The selenium-75-homocholic acid taurine test reevaluated: combined measurement of fecal selenium-75 activity and 3 alpha-hydroxy bile acids in 211 patients. *J. Nucl. Med. Off. Publ. Soc. Nucl. Med.* 32, 1219–1224.

Vanlier, J., Tiemann, C. A., Hilbers, P. a. J., and van Riel, N. a. W. (2012). An integrated strategy for prediction uncertainty analysis. *Bioinforma. Oxf. Engl.* 28, 1130–1135. doi:10.1093/bioinformatics/bts088.

Vanlier, J., Tiemann, C. A., Hilbers, P. A. J., and van Riel, N. A. W. (2013). Parameter uncertainty in biochemical models described by ordinary differential equations. *Math. Biosci.* 246, 305–314. doi:10.1016/j.mbs.2013.03.006.

Veysey, M. J., Malcolm, P., Mallet, A. I., Jenkins, P. J., Besser, G. M., Murphy, G. M., et al. (2001a). Effects of cisapride on gall bladder emptying, intestinal transit, and serum deoxycholate: a prospective, randomised, double blind, placebo controlled trial. *Gut* 49, 828–834. doi:10.1136/gut.49.6.828.

Veysey, M. J., Thomas, L. A., Mallet, A. I., Jenkins, P. J., Besser, G. M., Murphy, G. M., et al. (2001b). Colonic transit influences deoxycholic acid kinetics. *Gastroenterology* 121, 812–822. doi:10.1053/gast.2001.28015.

Vlahcevic, Z. R., Bell, C. C., Gregory, D. H., Buker, G., Juttijudata, P., and Swell, L. (1972a). Relationship of Bile Acid Pool Size to the Formation of Lithogenic Bile in Female Indians of the Southwest. *Gastroenterology* 62, 73–83. doi:10.1016/S0016-5085(72)80011-8.

Vlahcevic, Z. R., Juttijudata, P., Bell, C. C., and Swell, L. (1972b). Bile Acid Metabolism in Patients with Cirrhosis. *Gastroenterology* 62, 1174–1181. doi:10.1016/S0016-5085(72)80086-6.

Yanagisawa, J., Itoh, M., Ishibashi, M., Miyazaki, H., and Nakayama, F. (1980). Microanalysis of bile acid in human liver tissue by selected ion monitoring. *Anal. Biochem.* 104, 75–86.

Yuan, W., Zhang, Z., Liu, J., Li, Z., Song, J., Wu, C., et al. (2012). Simplified assessment of segmental gastrointestinal transit time with orally small amount of barium. *Eur. J. Radiol.* 81, 1986–1989. doi:10.1016/j.ejrad.2011.05.040.
