# Supplementary material for: In Silico Analysis Identifies Intestinal Transit as a Key Determinant of Systemic Bile Acid Metabolism
Source: Front Physiol. 2018 Jun 8;9:631. doi: 10.3389/fphys.2018.00631 (PMC6008656; doi:10.3389/fphys.2018.00631)
Supplement: Supplementary file 2 [file Data_Sheet_2.zip › BA_model_package/README for BA model package.docx]

README for BA model package

This readme is included in the supplementary material for: “In silico analysis identifies intestinal transit as a key determinant of systemic bile acid metabolism”

**Fianne L. P. Sips, Hannah M. Eggink, Peter A. J. Hilbers, Maarten R. Soeters, Albert K. Groen, Natal A. W. van Riel**

**Correspondence:** Fianne L.P. Sips: f.l.p.sips@tue.nl

- 1. **General introduction and necessary software**

This README file accompanies the *BA model* package, which was developed to run the bile acid model presented in the main text. The software of the *BA model* package accompanying this README file is provided as Matlab (MATLAB and Statistics Toolbox Release 2012a, The MathWorks, Inc., Natick, Massachusetts, United States) m-files. To perform parameter estimation as was done in this study, the optimization toolbox (we use version 6.2) should be installed. Computation of the model is expensive when Matlab functions (ode15s) are used for simulation. Therefore, the model is also provided such that it may be converted to MEX-files, via the SUNDIALS CVode package (2.6.0, Lawrence Livermore National Laboratory, Livermore, California) (Hindmarsh et al., 2005) and ODEMEX wrapper (Vanlier et al., 2012).

- 1. **Overview of the included files**

In order to run the model, the zip file *BA_model_package* must be unwrapped. In the main folder of the same name, you will now see the main run files, which can be called to recreate Figure 4 of the main text or perform an example simulation, respectively.

In addition to the files present in the main folder, which allow the user to run the model, there are a number of organized model files present. These files are grouped into the following folders:

- Calculations

*This folder contains small functions that are used for a variety of pre- and post- simulation calculations. The functions include several calculations of variables (composition by comp_human, conjugation by conj_human, postprandial variables by post_human) set up to return either a string of an equation for calculation of the variable, or the calculated value itself, depending on the value of input “equation”. To facilitate these calculations the functions eval_BA_list, eval_states_list, eval_sulf, eval_term and eval_tissue_list derive lists (of e.g. all BA species) or evaluate the presence of a certain species automatically, based on model structure. Finally, the folder contains several functions that derive postprandial characteristics (post_human, find_characteristics), that calculate the value of the transient postprandial functions (GI_reflex) and that calculate all plasma measures and the size of the DCA pool as used in the sensitivity and control analyses (plasma_measures).*

- Data

*This folder contains the excel file describing the CDS, as well as the functions necessary to extract and annotate the CDS.*

- Human_MST

*This folder contains the core model files and cost function. In addition to files used for simulation of the full model, there are also files included for two model variants: the minimal model used for the transit calculations and the extended model used for tracer simulations.*

- Results^[[1]](#footnote-1)^

*This folder contains the final optimization result, as well as a text file of model parameter values (and the m-files to generate this text file).*

- Simulation

*This folder contains all files used for simulation of the model. Note that simulation files are included for a full model simulation, as well as a transit simulation (to reduce computational costs, this is performed with a single bile acid). Also, files are included for an in silico cholecystectomy.*

- Vis Analysis

*This folder contains the file that is run to compose Figure 4.*

- 1. **Description of the optimization *result* structure**

All information necessary for model simulation and evaluation of the results is provided in the result structure. The structure contains fields that provide information on (a) data, (b) model settings, and (c) optimization results:

1. **Data.**

The *result* structure contains the data in two separate formats:

*CF_base* is a structure that is generated by *Data/retrieve_CD.m* with the aid of the calibration dataset excel file (*Data/* *20170317 Calibration_data_human.xlsx*). The structure contains not only the data points themselves, but also the information necessary to relate these data to model variables. Please see *Data/retrieve_CD.m* for additional information.

*datavec* is a vector that contains all 76 datapoints in vector format.

1. **Model/ optimization settings**

Both simulation (field: *model_info*) and optimization (field: *optim_options*) settings are contained in *result.* The *optim_options* structure is directly appropriate for use with *lsqnonlin*. The *model_info* structure is a custom structure, that contains all information necessary for simulation. An overview of the variables contained in model_info is provided in Table 1 below.

1. **Optimization results**

The *result* structure contains the outputs of lsqnonlin (*p_opt,resnorm,residual,exitflag,output,lambda,jacobian*). The value of *p_opt* guards the optimal parameter vector.

**Table 1. Contents of *model_info***

| ***Simulation settings*** | |
| --- | --- |
| doss | Days of “fasting” simulation ($n_{f}$): default of 20 |
| dors | Days of meal simulation ($n_{d}$): default of 35 |
| gap | Vector of the amount of time between. Note that a meal is simulated at the beginning of every time period. The default of [6 6 12] can be interpreted as breakfast, followed by a fast for 6 hours until lunch, followed by a fast for 6 hours until dinner, followed by an overnight fast of 12 hours. |
| steps | Number of steps recorded after each meal in the simulation. For the final 3 meals (= the final day), 50 * *steps* are recorded. |
| mex_settings | Integration settings used by the mex compiler. |
| ***Model constants***  *Note that in addition to the few model constants that have a value, the absence of a process or transformation is in many cases (re-)enforced by setting an associated constant to 0.* | |
| c | Structure containing lists of BA species and conjugation states, as well as constants. |
| c_loc | Location of individual constants in constant vector *c_vec.* |
| c_vec | Vector of values of constants. |
| ***Model parameters and bounds*** | |
| p | Structure defining (initial) parameter values. |
| l | Structure defining lower bounds for parameter values. |
| h | Structure defining upper bounds for parameter values. |
| p_loc | Structure relating parameter names with location in parameter vector *p_vec* |
| p_vec | Vector of (initial) parameter vectors |
| l_vec | Vector of lower bounds |
| h_vec | Vector of upper bounds |
| ***Model structure and design*** | |
| L | Number of state variables (including feces and cumulative states, that are not included in the base model) |
| o | Names of state variables in the order in which they are contained in the state vector. |
| ss | Vector to indicate whether the state variable is part of the base model. |
| data_loc | Vector relating the 76 data points to their location in the 34 data classes of CF_base. |
| ***Initial conditions*** | |
| x0 | Initial conditions of all state variables |
| ***Transit model*** | |
| L_tran | Number of state variables in the transit model. |
| o_tran | Location of state variables in the transit model. |
| x0_tran | Initial conditions of the transit model. |
| ss_tran | Vector to indicate whether the state variable is part of the base (transit) model. |
| c_tran | Constants of the transit model. |
| ***Handles for functions relating to model simulation*** | |
| h_ode_full | Right hand side of the differential equations, full model |
| h_var_full | Variables, full model |
| h_ode_tran | Right hand side of the differential equations, transit model |
| h_var_tran | Variables, transit model |
| h_c | Constants |
| h_p | Parameters |
| h_cf | Cost function |

- 1. **A short introduction to changing the simulation strategy**

To facilitate changing the simulation strategy, an example file is included in the main folder, which illustrates how to change to simulation strategy in the long-term, or simulate the long-term normally and change the simulation strategy acutely. Note that parameters can be changed either by accessing their index directly (Table 2, below), or by accessing their index through the structure *p_loc*, in which the index of each parameter can be found through the parameters name (Table 2).

Changes to model structure are more complex to implement, as they require changes to the *ode* and *var* files. If you wish to for instance adapt intestine length by skipping small intestinal compartments 3,4 and 5, this will require a change of the transit equations implemented in the model. Currently transports all bile acids in compartment 2 to compartment 3, which is implemented via fluxes of the following shape:

si_trans_out_u_LCA_2 = si2_u_LCA * k_si_trans_2_3;

and, reciprocally:

si_trans_in_u_LCA_3 = si2_u_LCA * k_si_trans_2_3;

A change of model structure as suggested above can be achieved by adaptation of all fluxes into compartment 3 to 0, and adaptation of all fluxes into compartment 6 as follows:

si_trans_in_u_LCA_3 = 0;

si_trans_out_u_LCA_2 = si2_u_LCA * k_si_trans_2_6;

si_trans_in_u_LCA_6 = si2_u_LCA * k_si_trans_2_6;

Where we note that the parameter k_si_trans_2_6 must also be defined. Furthermore, this change must be performed for all BA species.

**Table 2. Overview of parameters**

| **Location in p_vec** | **Name (code)** | **Name (manuscript)** |
| --- | --- | --- |
| 1 | GIr_beta_GB | $\beta_{GB}$ |
| 2 | GIr_beta_SI | $\beta_{SI}$ |
| 3 | GIr_delta_GB | $\delta_{GB}$ |
| 4 | GIr_delta_SI | $\delta_{SI}$ |
| 5 | k_xl | $k_{xl}$ |
| 6 | frac_gb | $\gamma_{GB}$ |
| 7 | k_xg | $k_{xg}$ |
| 8 | k_xi_up_si | $k_{xi,up,si}$ |
| 9 | k_xi_up_co | $k_{xi,up,co}$ |
| 10 | vmax_asbt_s | $V_{max,s}$ |
| 11 | vmax_asbt | $V_{max}$ |
| 12 | km_asbt | $K_{m}$ |
| 13 | frac_li_u | $\psi_{u}$ |
| 14 | frac_li_c_1 | $\psi_{tri}$ |
| 15 | frac_li_c_2 | $\psi_{di}$ |
| 16 | frac_li_c_3 | $\psi_{mono}$ |
| 17 | frac_li_s | $\psi_{sulf}$ |
| 18 | frac_tu | $\gamma_{t}$ |
| 19 | k_ut | $k_{ut}$ |
| 20 | k_ug | $k_{ug}$ |
| *21* | k_bact_dsi | $k_{bact}^{dsi}$ |
| *22* | k_si_1 | $k_{si,\alpha}$ |
| *23* | k_si_2 | $k_{si,\beta}$ |
| *24* | k_co_1 | $k_{co}$ |
| *25* | k_u | $k_{u}$ |
| *26* | frac_CA | $\gamma_{CA}$ |
| *27* | k_tr_1 | $k_{DCA, CA}$ |
| 28 | k_tr_2 | $k_{UDCA, CDCA}$ |
| *29* | k_tr_3 | $k_{LCA, CDCA}$ |
| *30* | k_tr_4 | $k_{LCA,UDCA}$ |
| 31 | k_tr_5 | $k_{LCA, LCAs}$ |
| 32 | k_tr_6 | $k_{LCAs,LCA}$ |
| 33 | k_tr_7 | $k_{oth,BA}$ |

- 1. **References**

Hindmarsh, A. C., Brown, P. N., Grant, K. E., Lee, S. L., Serban, R., Shumaker, D. E., et al. (2005). SUNDIALS: Suite of Nonlinear and Differential/Algebraic Equation Solvers. *ACM Trans Math Softw* 31, 363–396. doi:10.1145/1089014.1089020.

Vanlier, J., Tiemann, C. A., Hilbers, P. a. J., and van Riel, N. a. W. (2012). An integrated strategy for prediction uncertainty analysis. *Bioinforma. Oxf. Engl.* 28, 1130–1135. doi:10.1093/bioinformatics/bts088.

1. Beware of the difference between the *result* structure – containing the optimization result –and the structure referred to as e.g. healthy or *results* – containing the simulation results. [↑](#footnote-ref-1)
